# Patient and family perspectives on cascade screening for thoracic aortic disease: a mixed-methods evaluation

**DOI:** 10.1038/s41431-026-02051-8

**Published:** 2026-03-02

**Authors:** Riccardo Giuseppe Abbasciano, Joanne Miksza, Julian Barwell, Nora Shannon, Paul Clift, Riccardo Proietti, Una Ahern, Haleema Saadia, Gordon McManus, Kathryn Hewytt, Nadeem Qureshi, Laerke Ghosh, Ramanjit Kaur, Hardeep Aujla, Sue Page, Mark Lewis, Rob Sayers, Anne Cotton, Matt Bown, Lisa Skinner, John Maltby, George Krasopoulos, Duke Cameron, Aung Oo, John Elefteriades, Gareth Owens, Gavin James Murphy

**Affiliations:** 1 Department of Cardiovascular Sciences, University of Leicester, Leicester, UK; 2 Leicestershire Clinical Genetics Service, University Hospitals of Leicester NHS Trust, Leicester, UK; 3 Department of Clinical Genetics, Nottingham University Hospitals, Nottingham, UK; 4 Department of Cardiology, Queen Elizabeth Hospital Birmingham, Birmingham, UK; 5 Liverpool Centre for Cardiovascular Science, University of Liverpool and Liverpool Heart and Chest Hospital, Liverpool, UK; 6 Aortic Dissection Awareness UK & Ireland, Woking, UK; 7 Department of Cardiothoracic Surgery at University Hospitals Birmingham NHS Trust, Birmingham, UK; 8 NIHR School of Primary Care Research, University of Nottingham, Nottingham, UK; 9 Department of Cardiothoracic Surgery, Barts Heart Centre, St Bartholomew’s Hospital, London, UK; 10 Department of Cardiothoracic Surgery, John Radcliffe Hospital, Oxford, UK; 11 Department of Vascular Surgery, University Hospitals of Leicester NHS Trust, Leicester, UK; 12 School of Healthcare, University of Leicester, Leicester, UK; 13 Department of Cardiothoracic Surgery, The Johns Hopkins Hospital, Baltimore, MD USA; 14 Department of Cardiothoracic Surgery, Yale University School of Medicine, New Haven, CT USA

**Keywords:** Genetic services, Human behaviour

## Abstract

Cascade screening enables effective secondary prevention and early treatment for Thoracic Aortic Disease (TAD) and increases survival. Despite guideline recommendations, the uptake of screening remains low. This study investigated individual and organisational barriers to screening participation. We performed clinician and public focus groups (*n* = 19 participants across 5 sessions), semi-structured interviews (4 clinicians) and a national patient/relative survey (*n* = 242 responses: 71 probands, 171 relatives). Behavioural theories guided data interpretation and thematic analysis. Data collection explored motivations, psychological and practical burdens, communication dynamics and attitudes towards screening and Decision Support Tools (DSTs). A national survey of TAD patients and their families provided quantitative context on demographics, genetic testing uptake and involvement in shared decision-making. Thematic analysis using the framework approach was applied to qualitative data. Qualitative analysis of focus groups, interviews and a national patient/relative survey (*n* = 242) identified significant barriers to TAD cascade screening, including fragmented services, inconsistent clinician knowledge and patient confusion regarding genetic testing pathways. Survey data showed low genetic testing uptake (47% survivors; 44% and 21% for first- and second-degree relatives). Conversely, key facilitators for a DST included user-friendliness, multi-modal accessibility, clear risk/benefit communication and the inherent value of reassurance with professional endorsement from healthcare providers. These would directly address observed psychological and practical burdens. Patient and family engagement in TAD cascade screening faces complex barriers, including psychological burdens and systemic issues, resulting in a substantial shared decision-making gap. User-centric, multi-modal Decision Support Tools, supported by enhanced clinician education and structured family communication, are vital for effective TAD prevention

## Introduction

Thoracic Aortic Disease (TAD) is often characterised by a prolonged latent phase of asymptomatic aneurysm formation that often results in acute aortic dissection, an event with a mortality rate exceeding 70% [1, 2]. This condition affects approximately 1 in 2000 individuals and is responsible for nearly 3000 deaths annually in the UK, surpassing the mortality rate from road traffic accidents [3]. Over 20% of TAD cases have an identifiable genetic cause, with up to 30% of first- and second-degree relatives potentially carrying pathogenic variants or having asymptomatic aneurysms detectable through cascade genetic screening [4].

Clinically, TAD is classified into syndromic and non-syndromic forms (NS-TAD) [5]. Syndromic forms are associated with systemic connective tissue disorders such as Marfan, Loeys-Dietz and Vascular Ehlers-Danlos syndromes, where the yield of genetic testing is high and diagnosis is often aided by extra-aortic manifestations. Conversely, NS-TAD presents with isolated aortic involvement. While up to 20% of NS-TAD patients exhibit a family history, a pathogenic variant is currently identified in only 20-30% of these familial cases [6].

This condition carries a substantial psychological burden, with affected individuals reporting lower health-related quality of life compared to the general population. Notably, younger and female patients demonstrate lower health-related quality of life and higher depression scores than older male patients [7]. The familial clustering of this disease means first-degree relatives of affected individuals face elevated aortic-specific mortality, emphasising the catastrophic potential of latent disease within families.

Early detection of TAD through targeted cascade screening (both through imaging tests and genetic tests) of the families of those affected reduces mortality, as it enables timely secondary prevention and early treatment [1, 2]. Cascade genetic testing provides a definitive risk assessment but is only feasible when a pathogenic variant is identified in the proband (the first identified case in a family). When the proband is gene-negative or no variant of pathogenic significance is found, guidelines recommend that first-degree relatives undergo serial aortic imaging surveillance to detect phenotypic expression of the disease. International treatment guidelines recommend cascade screening involving genetic testing and aortic imaging for relatives of all people with Syndromic-TAD, with screening restricted to relatives of individuals with NS-TAD when the proband is under 60 years of age or has a family history of the disease [1, 2]. Despite these recommendations, the real-world availability and uptake of cascade screening is low and demonstrates regional variation [6, 8, 9].

Decision Support Tools (DSTs) have proven effective in bridging similar gaps in other medical fields, such as cancer screening and Familial Hypercholesterolemia, by translating complex guidelines into accessible, personalised risk assessments [10]. These tools facilitate shared decision-making by clarifying options and aligning medical recommendations with patient values [11].

Qualitative research methods are used to understand and overcome barriers to the implementation of healthcare interventions. The application of established behavioural science models can provide a structured mechanism for comprehending complex motivations and deterrents and analyse factors dictating professional behaviour [12]. These frameworks enable the interpretation of qualitative findings concerning patient attitudes and perceived control, providing structured explanations for the acceptance of both genetic screening and digital Decision Support Tools.

The aims of this study were to use established behavioural science models to investigate patient, family and clinical perspectives on TAD cascade screening, to identify key motivations, individual and institutional barriers and potential facilitators for engagement, using a mixed-method approach including focus groups, interviews and a survey. A specific objective was to inform the co-production of a Decision Support Tool (DST) that would enable informed, personalised, shared decisions about screening for probands and families [13].

## Materials and methods

### Design and participants

**Fig. 1:**
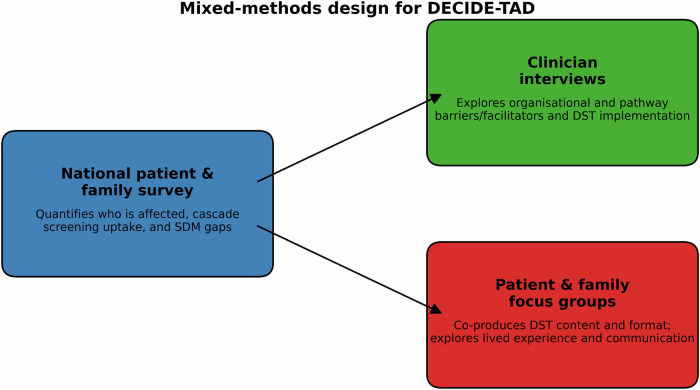
Overview of mixed-methods design and role of each component. This mixed-methods design included three distinct but complementary components. The specific role of each method was as follows: *National Patient and Family Survey* (Quantitative Context): A cross-sectional survey was administered to aortic dissection survivors and their relatives to quantify current screening uptake rates and statistically map demographic and pathway-related barriers across the target population. *Focus Groups* (User Experience): Qualitative sessions with patients and family members were conducted to explore the psychological nuances of these barriers—specifically regarding family communication—and to iteratively evaluate the functional requirements of a DST. *Clinician Interviews* (Implementation Feasibility): Semi-structured interviews with aortic specialists investigated the feasibility of implementing such interventions within the NHS, focusing on organizational constraints, workflow integration and professional acceptance. DST Decision Support Tool, NHS National Health Service, SDM Shared Decision Making.

This study used a mixed-methods, applied health research design. A national survey was administered to aortic dissection survivors (probands) and their relatives, providing a quantitative context to the lived experiences of TAD sufferers and their families. Semi-structured interviews and focus groups involving both clinicians and people with TAD and their family members were also performed. The research followed the Standards for Reporting Qualitative Research checklist [14]. (Fig. 1) [Supplementary Materials] Co-production involved a partnership with the national patient charity for aortic dissection survivors and the families of those with TAD; Aortic Dissection Awareness UK and Ireland (ADA-UKI).

The target population for this research was individuals at risk of NS-TAD, specifically aortic dissection survivors and their relatives and caregivers. Clinicians involved in providing care to these patients were also approached. Participants in focus groups and interviews were identified by the patients’ charity; the eligibility criteria were having experienced aortic dissection as a survivor or as a family member (members of the public). Clinicians were selected among those caring for people with TAD who had previously collaborated with the charity.

The Leicester Centre for Ethnic Health Research was involved in the project to help ensure that a diverse group of patients and relatives would be included in the focus groups. They provided a community engagement officer to advise researchers and the members of ADA-UKI who were recruiting participants. They also offered training to ADA-UKI members and attended a focus group to help facilitate the discussion.

### Methodological framework

The interpretation and analysis of the qualitative data drew on four established behavioural science frameworks: the Unified Theory of Acceptance and Use of Technology (UTAUT and its expansion UTAUT2) [15, 16], the Theory of Planned Behaviour (TPB) [17, 18], Self-Determination Theory (SDT) [19, 20] and Health Literacy models [21]. Rather than relying on a single model, which risks providing only a partial or skewed account of behaviour, these four frameworks were applied together to capture different but interconnected processes relevant to technology adoption and health-related decision-making, linking social, cognitive, motivational and informational dimensions. Each framework brings a distinct perspective, yet they often overlap in aspects of perception, intention, motivation and capability, reflecting the complex and dynamic ways in which these broader processes interact. The UTAUT and UTAUT2 examine technology adoption through factors such as performance and effort expectancy, social influence, facilitating conditions, hedonic motivation, price value and habit, focusing on users’ perceptions and interactions with technology. The TPB highlights how attitudes, subjective norms and perceived behavioural control shape intentions and subsequent behaviour. The SDT explains why individuals engage in specific behaviours and the extent to which these are self-determined, emphasising the importance of autonomy, competence and relatedness in fostering intrinsic motivation, well-being and engagement. Finally, Health Literacy models foreground individuals’ ability to access, understand, evaluate and use health information, which is crucial for making informed health decisions, particularly in technology use. Taken together, these frameworks provide a more comprehensive and multi-layered interpretation of the qualitative data than any single model alone.

### Analysis

A systematic thematic analysis approach was applied to the qualitative data to identify, analyse and report recurring patterns and themes [22]. Initial coding was conducted independently by two researchers, with disagreements resolved through discussion and consensus. Themes were developed inductively from the data while being informed by existing literature on genetic testing decision-making. Survey data were analysed descriptively, with findings integrated with qualitative themes to provide a comprehensive understanding of the phenomenon. To guide the interpretation of qualitative data, we considered the aforementioned theoretical contexts and therefore, the analysis specifically looked for:

- Perceptions and Usability of Technology: How participants viewed the ease of use and usefulness of genetic screening (intended as the genetic component of a cascade screening offer) technologies and DST.
- Behavioural Intentions: Attitudes towards genetic screening, including perceived benefits and risks.
- Social Influences: The impact of societal norms and opinions of others on participants’ decisions.
- Control and Accessibility: Participants’ perceptions of their ability to access and effectively use genetic screening technologies.
- Health Literacy Levels: How well participants understood genetic screening information and its implications.

Therefore, the analysis proceeded by initially classifying data deductively based on the research questions derived from these frameworks, followed by an iterative inductive modification and merging of the interpretation of the themes, both for the focus groups’ and interview data.

### Research procedures

The mixed methods exercise initiated with a national survey disseminated among members of Aortic Dissection Awareness UK & Ireland, followed by two rounds of focus groups with patients and relatives, along with focused interviews with clinicians. (Fig. 1).

A digital survey, consisting of 60 non-validated questions, was developed and administered via an online platform (SurveyMonkey, Symphony Technology Group, San Mateo, California) to members of Aortic Dissection Awareness UK & Ireland. A team of clinicians and patient-as-researchers produced the first draft of the survey, which underwent subsequent rounds of refinements in both content and structure with public involvement. Its primary purpose was to provide quantitative context on demographics, genetic testing uptake and reported involvement in shared decision-making among individuals at risk of TAD and to identify barriers to cascade screening to inform the design of a DST to support screening decisions. The survey was active for 7 months, from April to November 2022.

Two rounds of focus groups were conducted between October 2022 and May 2023. The first round evaluated participants’ opinions on the design and potential utility of a DST for cascade screening, building upon initial responses gathered from the national survey. The second round explored individual and institutional barriers to DST use, with a particular emphasis on challenges related to technology adoption. Sessions lasted approximately 90 min and were conducted using video conferencing to allow for broader geographical representation.

To complement the patient and public perspectives, clinicians involved in TAD management were invited to participate in focused interviews. These interviews explored the same topics as the focus groups, specifically aiming to identify and discuss strategies for overcoming existing barriers to screening and DST adoption. Interviews used open-ended questions.

Questionnaires and topic guides were created for the clinicians and the patients and family members and provided a structure for the focus groups and interviews [Supplementary Materials]. For patients and relatives, questions addressed the perceived importance of improved health outcomes, usability improvements, control over processes, data security and accessibility of information. Data processing prior and during the analysis included contemporaneous structured summary notes with full anonymization and de-identification and general information about the group’s composition was recorded.

## Results

### Survey findings

**Fig. 2:**
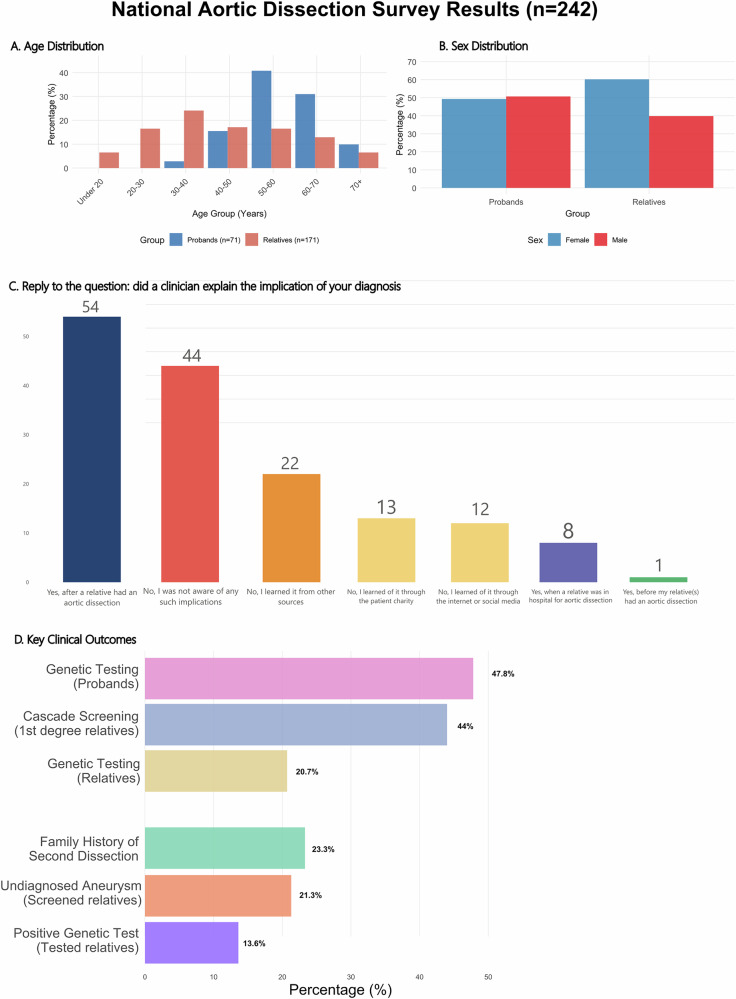
Summary of the demographic findings and key outcomes from the national survey. The chart summarises the age distribution (**A**) sex distribution (**B**) modalities with which participants learned about the screening implications connected to thoracic aortic disease (**C**) and key clinical outcomes (**D**) emerging from the national patient and relatives survey.

The national survey compiled 242 responses, derived from 71 aortic dissection survivors (probands) and 171 relatives. The demographic characteristics of these participants are detailed in Table 1 and in Fig. 2 along with the key findings from the survey. Results are also available in their entirety in the Supplementary Materials.

**Table 1:** Demographics of national patient survey participants.

	*Patients*	*Relatives*
**Number**	71	171
Age (Years) Mean (IQR)	58.4 (46.0–70.8)	42.5 (31.3–57.6)
Age (Years) categorised (%)		
Under 20	0 (0)	11 (6.5)
20–30	0 (0)	28 (16.5)
30–40	2 (2.8)	41 (24.1)
40–50	11 (15.5)	29 (17.1)
50–60	29 (40.8)	28 (16.5)
60–70	22 (31.0)	22 (12.9)
70+	7 (9.9)	11 (6.5)
Sex (%)		
Male	36 (50.7)	68 (39.8)
Female	35 (49.3)	103 (60.2)
Ethnicity (%)		
White	69 (97.2)	165 (95.9)
Black	1 (1.4)	1 (0.6)
Asian	0 (0.0)	1 (0.6)
Chinese	1 (1.4)	0 (0.0)
Mixed/Other	0 (0)	5 (2.9)
Region (%)		
Cymru/Wales	4 (5.9)	7 (4.1)
East	5 (7.4)	8 (4.7)
East Midlands	7 (10.3)	17 (10.1)
London	7 (10.3)	18 (10.7)
North East	2 (2.9)	6 (3.6)
North west	4 (5.9)	19 (11.2)
Scotland	2 (2.9)	4 (2.4)
South East	13 (19.1)	7 (4.1)
South West	13 (19.1)	35 (20.7)
West Midlands	3 (4.4)	24 (14.2)
Yorkshire and the Humber	8 (11.8)	11 (6.5)
Time since aortic dissection (%)		
One year	17 (23.9)	na
Two years	7 (9.9)	na
Three years	12 (16.9)	na
More than three years	35 (49.3)	na
Relation to person with dissection (%)		
First degree	na	154 (89.0)
Second degree	na	16 (9.3)
Other	na	3 (1.7)
Diagnosed with syndromic condition (%)		
Yes	6 (8.5)	5 (3.1)
No	65 (91.5)	159 (97.0)
Had a relative diagnosed with a syndromic condition (%)		
Yes	6 (8.5)	na
No	58 (81.7)	na
Don’t know	7 (9.9)	na
Received genetic testing (%)		
No, but was offered it	3 (2.9)	3 (1.9)
No, wasn’t offered it	34 (49.3)	120 (77.4)
Yes	33 (47.8)	32 (20.7)
Received genetics appointment (%)		
Yes	na	15 (9.2)
No	na	143 (87.7)
Not sure/ Don’t want to say	na	5 (3.1)

Demographics of all respondents to the national survey.

*Na* not applicable, *SD s*tandard deviation.

Genetic screening uptake revealed a marked gap in clinical initiation, as 49.3% of probands stated they were not offered testing, despite 47.8% ultimately receiving it. Only 20.7% of relatives had undergone genetic testing. This deficit contributed to substantial dissatisfaction regarding information provision and shared decision-making: 65.08% of probands and 75.20% of relatives reported feeling inadequately informed or excluded from decision processes related to their care. A widespread lack of primary care support was also observed, with 60.32% of probands and 79.03% of relatives indicating that their General Practitioner offered no information on relevant topics such as lifestyle management or family implications.

Willingness to use digital interventions was high, with 96.83% of probands and 86.26% of relatives agreeing to submit supplementary trial data via a mobile phone application. Preferences for receiving shared decision-making information showed flexibility: among probands, preferences were split between booklets (38.10%), websites (30.16%) and mobile apps (26.98%). Relatives prioritised websites (32.00%) and mobile apps (31.20%), slightly over booklets (29.60%).

In terms of logistical commitment captured through open responses, willingness to dedicate time for app usage varied significantly, with answers ranging from zero to 360 min per week, although most respondents cited 30 or 60 min. Similarly, the distance respondents were willing to travel for screening ranged broadly, extending from 0 miles to over 200 miles, with some relatives expressing willingness to travel nationally or ‘anywhere if it helps’.

Prioritising information for affected families (on a 5-point weighted scale, 5 = Essential) highlighted a high demand for clarity regarding definitive outcomes. The highest importance was placed on understanding the meaning of a positive genetic test result (4.24), followed closely by knowing how treatment effectiveness is confirmed (4.14), the consequences of treatment (4.13) and the significance of a positive imaging test (4.13).

Regarding access to a Decision Support Tool containing patient data, probands favoured Cardiologists (90.48%) and Surgeons (82.54%) having access, with high support also given to General Practitioners (87.30%) and Clinical Geneticists (80.95%).

Sixty-six out of 150 (44%) and 32/155 (21%) of the first- and second-degree relatives of TAD sufferers who responded were offered imaging or cascade genetic testing, respectively. When asked if anyone in their family was diagnosed with TAD, 32/149 (21%) replied that other members of their family were found to have an undiagnosed aneurysm via imaging and 20/147 (13.6%) replied that there was a positive genetic test in their family. Four out of 70 (6%) dissection survivors and 35/150 (23.3%) relatives who responded had a second family member who went on to have an aortic dissection. Only 19/71 (27%) probands and 20/155 (13%) relatives in our survey reported that they were involved in shared decisions about their care.

The quantitative results from the Survey are summarised and linked to the methodological frameworks' subdomains in Table 2.

**Table 2 -:** Alignment of quantitative survey results with elements of the theoretical framework.

Survey Finding (Quantitative Result)	Corresponding Thematic Result/Finding	Relevant Theoretical Construct
96.83% of probands and 86.26% of relatives were willing to submit supplementary data via a mobile phone application.	High willingness to adopt and use digital health technology.	**Performance Expectancy (UTAUT):** Participants believe the technology will be useful in achieving health goals. **Attitude toward the Behaviour (TPB):** Overall positive evaluation of engaging with the tool.
65.08% of probands and 75.20% of relatives reported inadequate information or exclusion from shared decision-making.	Significant deficiency in current decision support and information transfer.	**Health Literacy:** Inability to access, understand, evaluate or use relevant health information. **Autonomy (SDT):** Lack of involvement compromises self-determined decision-making.
49.3% of probands stated they were not offered genetic testing.	A failure in systematic care delivery prevents access to genetic testing.	**Facilitating Conditions (UTAUT):** Lack of necessary resources or organizational support to complete the process smoothly. **Perceived Behavioural Control (TPB):** External barriers inhibit the ability to perform the desired behaviour.
Relatives reported high familiarity with digital devices (87.90% Very Familiar with mobile phones).	High baseline digital confidence among users.	**Effort Expectancy (UTAUT):** Perceived ease of use of technology is high. **Competence (SDT):** Confidence in their ability to engage with the tool successfully.
Information prioritizing highest importance scores (on a 5-point scale) was: meaning of a positive genetic test (4.24) and treatment effectiveness confirmation (4.14).	Strong need for clarity regarding outcomes and intervention efficacy.	**Health Literacy:** Requirement for understandable and evaluated health information to make informed decisions. **Performance Expectancy (UTAUT):** Focus on knowing the definitive benefit of screening results.
Cardiologists (90.48%), General Practitioners (87.30%) and Surgeons (82.54%) were highly favoured by probands for having access to the Decision Support Tool.	Strong reliance on key healthcare professionals for endorsement and guidance.	**Social Influence (UTAUT):** Belief that important individuals think they should use the technology. **Subjective Norms (TPB):** Influence of medical professionals reinforcing the behaviour.
Email was the primary preferred method for future research involvement for both probands (37.93%) and relatives (49.59%).	Preference for specific, accessible communication channels.	**Effort Expectancy (UTAUT):** Preference for low-effort, convenient communication. **Facilitating Conditions (UTAUT):** Identified infrastructure (email) that supports engagement.

Top of Form

Bottom of Form

Correlation between selected quantitative findings derived from the national survey with established constructs from the Unified Theory of Acceptance and Use of Technology (UTAUT/UTAUT2), the Theory of Planned Behaviour (TPB), Self-Determination Theory (SDT) and Health Literacy models.

### Thematic analysis

Nineteen participants, including aortic dissection survivors and their family members, engaged in five focus group sessions. Among those who provided demographic information, 4/12 (42%) were female and 5/12 (83%) were of White ethnicity, 1 (8%) was from Asian and 1 (8%) was from Caribbean backgrounds. Participants were younger (mean age: 58.4) than the UK average TAD patient population (73.0, IQR 63.0–81.0).

Four clinicians involved in the management of thoracic aortic disease participated in focused interviews and the analysis was guided by the Unified Theory of Acceptance and Use of Technology, the Theory of Planned Behaviour, Self-Determination Theory and Health Literacy models.

#### Perceptions and usability of technology

*Performance Expectancy* was high. Participants generally held positive attitudes towards the use of a DST with technology, expressing a high likelihood that the DST would yield positive outcomes and improve care, for example, by consolidating all relevant information in one place, providing access to reliable resources and offering anytime accessibility. However, concerns were raised regarding the clarity of information provided within the DST, with a risk of misinterpretation that could lead to misunderstandings. Specifically, participants identified complex genetic outcomes as challenging, including the implications of false positive, false negatives and inconclusive results. A debate occurred over whether to include statistics relating to these false results, as some participants felt this quantitative data would be excessively difficult to understand. Participants suggested that clinicians could help verify understanding after DST use. *Effort Expectancy* or the perceived ease of use, was a notable theme. While many found accessing digital content online straightforward, some reported difficulty using applications due to health conditions. Concerns about equitable access were prominent, particularly for older patients, non-English speakers and individuals with varying digital literacy or disabilities. Suggested improvements included multilingual support, provision of downloadable information and leaflets and the use of simple language and visual aids. One participant raised the potential financial or logistical burden of requiring specific technology, such as iPads, suggesting hospital provision to mitigate this barrier.

Clinicians emphasised the importance of user-friendly and universally accessible DSTs available in varied formats. While not explicitly stated as ‘hedonic motivation’, suggestions for user-specific content tailoring and ease of navigation hinted at a desire for a more enjoyable and satisfying user experience.

#### Behavioural intentions

*Attitude toward the Behaviour* (TPB) was positive, strongly motivated by altruism, specifically the desire to protect family members, particularly children, by identifying genetic susceptibility and enabling preventative measures. Participants expressed a general positive attitude towards genetic testing, with some reporting no perceived risks. They also sought genetic testing to understand the aetiology of their own TAD. While overall intentions for genetic screening were proactive, the lack of clear information regarding the pathway to testing indicated incomplete implementation plans.

#### Social influences

The *impact of Social Influence* (UTAUT) and *Subjective Norms* (TPB) on screening decisions was evident. While most participants were open to recommending the DST to family members, there were mixed feelings about whether family members would accept genetic screening or use the DST themselves. The impact of Subjective Norms (TPB) on screening decisions was evident, as some family members displayed an unwillingness to engage in testing discussions. Specifically, certain relatives declined screening due to a desire to avoid knowing their risk status, along with concerns about the potential impact on employment and insurance or a desire to avoid knowing their risk status.

Clinicians highlighted the importance of professional endorsement to increase trust and acceptance of the DST among patients.

#### Control and accessibility

Participants frequently expressed a sense of low *Perceived Behavioural Contro*l (TPB) for cascade screening. This was attributed to perceptions of the genetic screening process as difficult to navigate and a perceived knowledge deficit among General Practitioners (GPs) regarding TAD and genetic testing further hindered patient progression. Many participants reported actively pursuing access to screening and results themselves due to these barriers. They suggested that a DST would only be useful if existing barriers to genetic testing were addressed, as even motivated patients currently encountered difficulties. Facilitators included the *Social Influence* component of UTAUT, which regards healthcare professionals’ effect on user conduct and the *Competence* component of SDT, helped by information and aid. Clinicians recommended that the DST should be made convenient and easy to use through various delivery modes and personalised content, thereby enhancing users’ perceived control. Furthermore, concerns about data privacy and the need for transparent data collection and usage practices were emphasised as crucial for decision-making and trust. They also suggested that the DST should be normalised and integrated into the genetic counselling process, reflecting professional norms around its usage.

Clinicians generally foresaw DST adoption leading to better patient care, improved patient information, more efficient consultations and more direct job roles. While acknowledging the time and instruction needed for DST skills, they saw the tool as largely fitting with current job duties. Key implementation methods identified by clinicians included clear plans for stakeholder recognition, management backing, full instruction and smooth DST fitting into current care paths. Adding elements such as a decision-making chatbot or encouraging family involvement were also seen as useful additions, strengthening *Aid Conditions* and *Relatedness*, respectively.

#### Health literacy

*Facilitating Conditions*, UTAUT and low *Autonomy*, SDT were identified as barriers to uptake. Participants struggled to access accurate and useful information about genetic testing and TAD, indicating a general lack of clarity and prompting many to undertake their own research. This suggests a barrier to the ‘accessing’ and ‘understanding’ components of health literacy. While participants appreciated the convenience of having all information in one place via the DST, concerns about potential misinterpretation were raised. Despite these challenges, participants were largely engaged in evaluating the advantages and disadvantages of using a DST, highlighting its potential role in promoting health and prevention by providing information about genetic screening and helping them understand their risks (*Performance Expectancy*, UTAUT).

Clinicians also underlined the DST’s role in enhancing understanding for patients and families by providing essential genetic testing information and aiding decision-making. They further noted concerns about disparities in DST adoption among different groups within the National Health Service (NHS) and highlighted the need for effective training to ensure accurate use and understanding of health information across diverse literacy levels.

Illustrative quotes from are provided and linked to the methodological frameworks subdomains in Table 3.

**Table 3 -:** Illustrative quotes aligned with theoretical frameworks.

Framework	Sub-Domain	Theme Description	Illustrative Quotation
TPB	Attitude toward Behaviour	Participants hold a strong belief in the effectiveness of genetic testing to identify susceptibility and enable preventive measures, thereby helping protect family members.	*I was not offered a test but proactively sought help through my doctor and ongoing referrals to get to a genetics test*. *I had to fight to get an appointment and only knew to because of the Facebook buddies group*
TPB	Subjective Norms	The influence of healthcare professionals (such as specialist aortic nurses or geneticists) on decision-making is significant; patients prioritise discussions with trusted staff members.	*I broached the subject with one of my daughters, a registered nurse, who considered it unnecessary*.
TPB/UTAUT	Perceived Behavioural Control (PBC)	Concerns arise regarding the complexity and accessibility of the screening process due to perceived professional knowledge deficits among General Practitioners.	*Have been waiting 18 months for an appointment*. *GP referred me to the wrong department. Still waiting after 3 years*
TPB/Health Literacy	Perceived Behavioural Control (PBC)	Patient fears related to the potential misinterpretation of complex results, specifically false positive, false negatives and inconclusive outcomes, reduce confidence in decision-making.	*Worried about Army employment if a genetic mutation were found* *Told it wasn’t necessary because my tests didn’t highlight any issues*
SDT	Relatedness	The involvement of healthcare staff is considered essential and encouragement of family participation reinforces the importance of genetic results extending beyond the individual.	*I had a screening in London in the last 2 years at Guy's Hospital. MRI and ultrasound*. *The consultant did not recommend genetic screening for me, but to investigate my brother's DNA in more detail*.

Collection of direct quotes from study participants, correlated with theoretical frameworks and subdomains.

*UTAUT* unified theory of acceptance and use of technology, *TPB* theory of planned behaviour, *SDT* self-determination theory.

## Discussion

### Main findings and contextualisation with literature

This mixed-methods analysis revealed how, in the context of TAD, while patients and families demonstrate high Performance Expectancy and positive Attitudes to Behaviour regarding cascade screening, actual engagement is severely hindered by systemic and psychological barriers. Our survey results confirmed that screening uptake remains consistently low (below 50% for eligible probands) and reported involvement in shared decision-making is critically impoverished (27% for probands; 13% for relatives). Qualitative themes indicate that, despite high motivation, engagement is obstructed by low Perceived Behavioural Control and Autonomy, primarily driven by fragmented care pathways, insufficient health literacy support and the psychological burden of navigating complex genetic information without adequate Facilitating Conditions.

Our data corroborates recent findings by Longoni et al. [8] and DeHart et al. [9], who similarly identified significant disparities between guideline recommendations and the real-world implementation of genetic testing in cardiovascular cohorts. Specifically, the barriers we identified regarding fragmented care pathways echo the qualitative work of Devine et al. [23] in cancer screening, where patients frequently report the burden of managing their own care coordination amidst systemic inefficiencies.

The low uptake of guideline-directed genetic testing observed in our cohort aligns with broader trends in inherited cardiovascular conditions, where real-world utilisation frequently lags behind clinical recommendations [24, 25]. However, our application of behavioural frameworks elucidates why this gap persists. Under the UTAUT model [15, 16], Facilitating Conditions are a prerequisite for behaviour; our findings suggest that without clear clinical pathways, the burden falls on the patient to ‘chase’ care, effectively negating their positive intentions.

Furthermore, the anxiety regarding inconclusive results (Variants of Uncertain Significance) and insurance implications mirrors findings in other genetic screening pathways [24, 26], suggesting that the complexity of risk information exceeds current Health Literacy capabilities for many patients. This deficit in ‘understanding’ and ‘evaluating’ health information directly impacts Autonomy (SDT), leaving patients dependent rather than empowered. The strong participant preference for a DST to consolidate reliable information supports the evidence that decision aids can significantly reduce decisional conflict and improve Competence by structuring complex medical data into actionable knowledge. Unlike standard care, which participants described as fragmented, a co-produced DST addresses the Relatedness component of SDT by validating the family’s experience and providing the professional endorsement necessary to overcome social hesitations regarding family communication.

The process to produce such a DST is currently ongoing [13]. The approach is based on co-design, via a collaboration between end-users and clinicians. An implementation guide and training package will also accompany the DST itself to ensure consistency in its adoption. Finally, randomised evidence of effectiveness on a national level (and potentially refinements of the tool) will be sought before established rollout in clinical practice.

In inherited conditions like Familial Hypercholesterolemia, digital tools aid in patient-centered care and delivery of cascade testing.^23^ The primary motivation identified here—protection of family members—mirrors the core benefit cited by Familial Hypercholesterolemia index patients: predicting risk for relatives. This confirms that reinforcing Performance Expectancy (UTAUT) related to prevention is an influential aspect for DST adoption.

### Implications for clinical practice

First, clinicians require targeted training in narrative-based risk communication, moving beyond mere statistical presentation. This involves utilising visual aids, empathetic dialogue and plain language to bridge the observed communication mismatch between medical and lay understandings of risk. The focus should shift to considering the emotional and personal relevance of a diagnosis, ensuring information is not only accurate but also comprehensible and actionable for patients and their families [27, 28].

Second, the low reported rate of shared decision-making highlights the need to integrate these processes into routine clinical encounters. This involves clearly presenting all reasonable screening options, including their benefits and potential harms, actively eliciting patient and family values and preferences and ensuring that individuals fully understand their role in the decision-making process [29]. DST can significantly facilitate this by structuring information and guiding value clarification exercises [11].

Third, addressing barriers to cascade screening for TADS in primary care is essential. Clear, easy-to-navigate pathways for genetic testing, genetic counselling and follow-up must be established and widely communicated, both within specialist centres and to primary care providers. Reducing waiting times and alleviating the burden on patients to actively ‘chase’ information and appointments are essential steps to improve accessibility [5].

Fourth, care pathways must extend beyond the point of diagnosis or screening to include comprehensive post-screening information, clear long-term surveillance plans and accessible psychological support for managing uncertainty or positive results. Resources considering non-clinical concerns, such as the potential impact of a diagnosis on employment and insurance, should also be readily available to alleviate patient anxieties [23]. DSTs are perceived as powerful instruments capable of bridging the gap between complex medical information and patient understanding, thereby promoting informed decision-making and empowering patients and families. These tools can standardise information delivery, ensure consistency across providers and serve as a centralized platform for providing comprehensive and holistic resources.

### Strengths and limitations

The application of established behavioural theories provides a robust framework for interpreting the complex interaction of factors influencing engagement. The inclusion of perspectives from both patients/relatives and clinicians provides a comprehensive view of the challenges and opportunities within the cascade screening pathway. Furthermore, the survey provides corroboration of the qualitative findings. Finally, co-production with a national patient charity for aortic dissection survivors and their families ensured the research was patient-centred and clinically relevant.

A limitation is the potential for selection bias in the qualitative samples, which included participants with higher health and digital literacy and a proportion of White ethnicity than the UK average. This demographic profile may limit the generalisability of some findings to less engaged or diverse populations, suggesting that the identified barriers might be even more pronounced in underserved communities. The reliance on self-reported survey data is also a consideration. The study also did not provide specific examples of geographic variation in services (which are the object of a separate analysis) or longitudinal follow-up data, which could offer further depth.

## Conclusion

Engagement with screening is hindered by systemic, informational and social challenges.

This study highlights the complex network of factors shaping cardiovascular screening and suggests how these insights can be translated into tangible improvements in practice, research and policy. Successful TAD prevention relies not only on increased clinical offer of interventions but also robust, sustained investment in enhanced clinician education and structured support for family communication. Both patients and clinicians see favourably the introduction of a DST to reach enhanced screening uptake and early detection, as well as a more equitable, autonomous and patient-centred approach to TAD prevention.

## Supplementary information

Supplementary Material
