# Supplementary material for: Patient and family perspectives on cascade screening for thoracic aortic disease: a mixed-methods evaluation
Source: Eur J Hum Genet. 2026 Mar 2;34(7):947–55. doi: 10.1038/s41431-026-02051-8 (PMC13342339; doi:10.1038/s41431-026-02051-8)
Supplement: Supplementary file 1 — Supplementary Material [file 41431_2026_2051_MOESM1_ESM.docx]

**Patient and Family Perspectives on Cascade Screening for Thoracic Aortic Disease: A Mixed-Methods Evaluation**

**Supplementary Materials**

Contents

[Questions for Patients and Families 3](#_Toc215987919)

[Questions for Clinicians 4](#_Toc215987920)

[National Survey Questions and Results 7](#_Toc215987921)

[Standards for Reporting Qualitative Research (SRQR)* 138](#_Toc215987922)

## Questions for Patients and Families

**DECIDE TAD Design of tool**

Ask everyone to briefly introduce themselves.

Briefly explain the wider project.

Give explanation and examples of what a decision support tools is.

Explain how the focus group will work and how to use Zoom chat etc.

**What information is important?**

- Risks/benefits
- False positives/negatives/inconclusive results
- What does testing involve?

**Who should the tool be aimed at?**

- Aortic dissection patients
- Family members 1^st^/2^nd^ degree
- How will family members access decision support tool?

**When should the tool be used?**

- In hospital after aortic dissection
- Outpatients
- Genetics/ cardiologist/ specialist nurse
- Available for considering at home

**How should information be communicated and what format should the tool take?**

- Diagrams/ infographics/written
- Pictures/ video
- App/ leaflets/ online
- Should it have any interactive elements
- Have links to information/ support groups

**Anything else that participants think should be discussed**

**Barriers to use**

**Introductions and explanations**

- Ask everyone to briefly introduce themselves
- Brief explanation of topics discussed in WP1 focus groups and formats tool could take.
- Explain purpose of this focus group.
- Explain how focus group will work/ zoom etiquette etc.

**Personality (innovation/risk)**

- What reasons would there be for you wanting to use the DST or reasons not to use the DST?
- How much are you interested in technology in DST? Discuss different possibilities apps/online info/interactive. Why/ why not.
- Would you be someone who would naturally use a DST?
- Would you be someone who would be happy to use technology as part of a DST? apps/online etc.

**Attitudes (trust/motivations)**

- How much do you trust technology? Use of data/accuracy.
- Do you think the DST will be useful? (Ways it is/ Is not)
- How would you feel about using the DST? (Concerns/ emotions)
- How likely would you be to use it? (how likely to use technology as part of it?)

**Social (subjective norms/self-image)**

- How would family members feel about using the DST?
- Would you be happy to recommend using a DST to family members? (problems/encourage them to have genetic screening).
- Do you think the use of technology would make it difficult for family members to use a DST?

**Cognitive (risk perception/uncertainty/familiarity/expertise/experiences)**

- Do you perceive any risks to using DST? (Do risks increase with inclusion of technology?)
- Do you perceive any risks with recommending DST to family?
- Do you see any problems with you/family members/patients being able to use DST/technology)

## Questions for Clinicians

**Organisational Issues**

1. Management/Leadership: How do you think the new technology implementation should be managed and led within the NHS?
2. What kind of leadership support do you think is necessary to facilitate successful implementation of the new technology?
3. How can leaders ensure that all staff members feel included and engaged throughout the implementation process?

**4**. Organisational Culture: How do you perceive the current organisational culture within the NHS in terms of openness to new technology adoption?

1. What changes, if any, do you think need to be made to the organisational culture to successfully integrate the new technology?
2. How can the NHS foster a culture of continuous improvement and learning to support ongoing technological advancements?

**7.** Rewards and Risks: What do you think are the main rewards and risks associated with implementing the new technology within the NHS?

1. How can the organisation ensure that the potential rewards outweigh the risks?
2. What risk mitigation strategies should be in place during the implementation process?

**10.** Job Fit: How well do you think the new technology aligns with the current roles and responsibilities within the NHS?

1. What changes, if any, to job roles do you anticipate as a result of implementing the new technology?
2. How can the NHS manage potential job displacement or adjustments related to technology implementation?

**13.** Compatibility with the Organisation: How compatible do you think the new technology is with the existing systems and processes within the NHS?

1. What modifications, if any, need to be made to ensure the seamless integration of the new technology?
2. How can the NHS prepare its infrastructure for successful technology integration?

**16.** Possibility of Adoption: How likely do you think it is that the new technology will be adopted successfully within the NHS?

1. What barriers to adoption do you foresee and how can they be overcome?
2. What factors are crucial for successful technology adoption in the NHS?

**19.** Expected Results: What are the expected benefits of implementing the new technology within the NHS?

1. How can the NHS measure the success of the new technology implementation?
2. What long-term impacts do you expect the new technology to have on patient care and overall organisational efficiency?

**Individual Practitioner Issues:**

1. Management/Leadership: As an individual practitioner, what kind of support do you need from management during the technology implementation process?
2. How can leaders effectively communicate the benefits and expectations of the new technology to individual practitioners?
3. What role should individual practitioners play in the decision-making process related to new technology adoption?

**4.** Organisational Culture: How does the current organisational culture within the NHS affect your willingness to adopt new technologies?

1. What changes to the organisational culture do you think would make you more open to embracing new technologies?
2. How can individual practitioners contribute to fostering a culture of continuous improvement and learning?

**7.**  Rewards and Risks: As an individual practitioner, what do you perceive as the main rewards and risks associated with the new technology?

1. How can you, as an individual practitioner, ensure that the potential rewards outweigh the risks when adopting new technologies?
2. What steps can you take to mitigate risks during the technology implementation process?

**10.** Job Fit: How do you feel the new technology will affect your role and responsibilities as an individual practitioner?

1. What additional training or support do you think you will need to adapt to the changes brought about by the new technology?
2. How can the NHS help individual practitioners manage potential job displacement or adjustments related to technology implementation?

**13.** Compatibility with the Organisation: How compatible do you think the new technology is with your current way of working within the NHS?

1. What adjustments, if any, do you think you will need to make to ensure the seamless integration of the new technology into your daily

## National Survey Questions and Results

*Patients*

Q1 Approximately how many years is it since your Aortic Dissection?

Answered: 71 Skipped: 0

| **ANSWER CHOICES** | **RESPONSES** |  |
| --- | --- | --- |
| About 1 year | 23.94% | 17 |
| About 2 years | 9.86% | 7 |
| About 3 years | 16.90% | 12 |
| More than 3 years | 49.30% | 35 |
| TOTAL |  | 71 |

Q2 In which region of the UK do you live?

Answered: 68 Skipped: 3

| **ANSWER CHOICES** | **RESPONSES** |  |
| --- | --- | --- |
| East | 7.35% | 5 |
| East Midlands | 10.29% | 7 |
| London | 10.29% | 7 |
| North East | 2.94% | 2 |
| North West | 5.88% | 4 |
| Northern Ireland | 0.00% | 0 |
| Scotland | 2.94% | 2 |
| South East | 19.12% | 13 |
| South West | 19.12% | 13 |
| Cymru/Wales | 5.88% | 4 |
| West Midlands | 4.41% | 3 |
| Yorkshire And The Humber | 11.76% | 8 |
| TOTAL |  | 68 |

Q3 What is your gender?

Answered: 71 Skipped: 0

| **ANSWER CHOICES** | **RESPONSES** |  |
| --- | --- | --- |
| Female | 49.30% | 35 |
| Male | 50.70% | 36 |
| Prefer not to say | 0.00% | 0 |
| TOTAL |  | 71 |

Q4 What is your current age (in years)?

Answered: 71 Skipped: 0

| Number | 71 |
| --- | --- |
| Age (Years) Mean (IQR) | 58.4 (46.0 - 70.8) |

Q5 Which ethnic group do you belong to?

Answered: 71 Skipped: 0

| **ANSWER CHOICES** | **RESPONSES** |  |
| --- | --- | --- |
| English, Welsh, Scottish, Northern Irish or British | 90.14% | 64 |
| Irish | 5.63% | 4 |
| Gypsy or Irish Traveller | 0.00% | 0 |
| Any other white background | 2.82% | 2 |
| White and Black Caribbean | 0.00% | 0 |
| White and Black African | 0.00% | 0 |
| White and Asian | 0.00% | 0 |
| Any other mixed or multiple ethnic background | 0.00% | 0 |
| Indian | 0.00% | 0 |
| Pakistani | 0.00% | 0 |
| Bangladeshi | 0.00% | 0 |
| Chinese | 1.41% | 1 |
| Any other Asian background | 0.00% | 0 |
| African | 0.00% | 0 |
| Caribbean | 1.41% | 1 |
| Any other Black, African or Caribbean background | 0.00% | 0 |
| Any other ethnic group | 0.00% | 0 |
| Other (please specify) | 0.00% | 0 |
| Total Respondents: 71 |  |  |

Q6 Had you ever heard about aortic dissection before your experience with it?

Answered: 70 Skipped: 1

| **ANSWER CHOICES** | **RESPONSES** |  |
| --- | --- | --- |
| Yes, from someone close to me | 11.43% | 8 |
| Yes, from healthcare professionals | 1.43% | 1 |
| Yes, from other sources | 1.43% | 1 |
| No, I had never heard about it | 85.71% | 60 |
| TOTAL |  | 70 |

Q7 Had you ever received specialist care for a cardiovascular condition (diseases of your heart and/or arteries)?

Answered: 70 Skipped: 1

| **ANSWER CHOICES** | **RESPONSES** |  |
| --- | --- | --- |
| Yes, I previously saw a specialist and I had a planned follow-up | 11.43% | 8 |
| Yes, I previously saw a specialist appointment, but I had no planned follow-up | 2.86% | 2 |
| No, I never received any specialist care for a cardiovascular condition | 85.71% | 60 |
| TOTAL |  | 70 |

Q8 Was your blood pressure within the normal range (as per your specialist’s or GP’s advice)?

Answered: 70 Skipped: 1

| **ANSWER CHOICES** | **RESPONSES** |  |
| --- | --- | --- |
| Yes, with medications | 25.71% | 18 |
| Yes, without need for medications | 22.86% | 16 |
| No | 24.29% | 17 |
| I don’t know | 27.14% | 19 |
| TOTAL |  | 70 |

Q9 Did you smoke?

Answered: 70 Skipped: 1

| **ANSWER CHOICES** | **RESPONSES** |  |
| --- | --- | --- |
| I never smoked | 52.86% | 37 |
| I smoked in the past, but I had stopped | 35.71% | 25 |
| I smoked | 11.43% | 8 |
| TOTAL |  | 70 |

Q10 What was your level of physical activity?

Answered: 70 Skipped: 1

| **ANSWER CHOICES** | **RESPONSES** |  |
| --- | --- | --- |
| Intense physical activity, for example, weightlifting in the gym | 5.71% | 4 |
| Fitness exercise and generally an active lifestyle | 71.43% | 50 |
| Sedentary lifestyle, for example, never walking more than 200 yards regularly | 22.86% | 16 |
| TOTAL |  | 70 |

Q11 Had you been diagnosed with any aortic syndrome such as Marfan, Ehlers-Danlos, Loeys-Dietz, or Osteogenesis Imperfecta (brittle bones)?

Answered: 69 Skipped: 2

| **ANSWER CHOICES** | **RESPONSES** |  |
| --- | --- | --- |
| Yes | 8.70% | 6 |
| No | 91.30% | 63 |
| TOTAL |  | 69 |

Q12 Did you ever have a Genetics appointment?

Answered: 70 Skipped: 1

| **ANSWER CHOICES** | **RESPONSES** |  |
| --- | --- | --- |
| Yes | 20.00% | 14 |
| No | 77.14% | 54 |
| I don’t know | 2.86% | 2 |
| I prefer not to say | 0.00% | 0 |
| TOTAL |  | 70 |

Q13 In the past, was there ever a sudden death among your relatives?

Answered: 70 Skipped: 1

| **ANSWER CHOICES** | **RESPONSES** |  |
| --- | --- | --- |
| Yes | 54.29% | 38 |
| No | 32.86% | 23 |
| I don’t know | 12.86% | 9 |
| I prefer not to say | 0.00% | 0 |
| TOTAL |  | 70 |

Q14 In the past, were there any known aortic aneurysms among your relatives, or did anyone previously require treatment for their aorta?

Answered: 70 Skipped: 1

| **ANSWER CHOICES** | **RESPONSES** |  |
| --- | --- | --- |
| Yes | 31.43% | 22 |
| No | 45.71% | 32 |
| I don’t know | 22.86% | 16 |
| I prefer not to say | 0.00% | 0 |
| TOTAL |  | 70 |

Q15 In the past, was there ever an aortic dissection among your relatives?

Answered: 70 Skipped: 1

| **ANSWER CHOICES** | **RESPONSES** |  |
| --- | --- | --- |
| Yes | 21.43% | 15 |
| No | 45.71% | 32 |
| I don’t know | 32.86% | 23 |
| I prefer not to say | 0.00% | 0 |
| TOTAL |  | 70 |

Q16 Were any of your relatives ever diagnosed with any syndrome, such as Marfan, Ehlers-Danlos, Loeys-Dietz, or Osteogenesis Imperfecta (brittle bones)?

Answered: 70 Skipped: 1

| **ANSWER CHOICES** | **RESPONSES** |  |
| --- | --- | --- |
| Yes | 8.57% | 6 |
| No | 81.43% | 57 |
| I don't know | 10.00% | 7 |
| I prefer not to say | 0.00% | 0 |
| TOTAL |  | 70 |

Q17 Did any of your relatives receive specialist care for cardiovascular conditions (diseases of the heart and arteries)?

Answered: 70 Skipped: 1

| **ANSWER CHOICES** | **RESPONSES** |  |
| --- | --- | --- |
| Yes | 52.86% | 37 |
| No | 37.14% | 26 |
| I don't know | 10.00% | 7 |
| I prefer not to say | 0.00% | 0 |
| TOTAL |  | 70 |

Q18 Did a healthcare professional ever tell you about the possible implications for your family due to the genetic nature of aortic dissection?

Answered: 70 Skipped: 1

| **ANSWER CHOICES** | **RESPONSES** |  |
| --- | --- | --- |
| Yes, before my aortic dissection | 2.86% | 2 |
| Yes, during my hospital stay for aortic dissection | 21.43% | 15 |
| Yes, during my follow-up after aortic dissection | 21.43% | 15 |
| No, I learned of it through the patient charity | 15.71% | 11 |
| No, I learned of it through the internet or social media | 8.57% | 6 |
| No, I learned it from other sources | 8.57% | 6 |
| No, I was not aware of any such implications | 21.43% | 15 |
| TOTAL |  | 70 |

Q19 Was any form of counselling or support arranged for you during your hospital stay or follow up?

Answered: 70 Skipped: 1

| **CHOICES** | **RESPONSES** |  |
| --- | --- | --- |
| Yes, and It helped | 17.14% | 12 |
| Yes, but it didn't help | 1.43% | 1 |
| No, but I think it would have helped | 62.86% | 44 |
| No, and I don't think it would have helped | 18.57% | 13 |
| TOTAL |  | 70 |

Q20 Did you have genetic testing?

Answered: 69 Skipped: 2

| **ANSWER CHOICES** | **RESPONSES** |  |
| --- | --- | --- |
| Yes | 47.83% | 33 |
| No, but I was offered it | 2.90% | 2 |
| No, I wasn't offered it | 49.28% | 34 |
| TOTAL |  | 69 |

Q21 Are you taking medications for your blood pressure?

Answered: 70 Skipped: 1

| **ANSWER CHOICES** | **RESPONSES** |  |
| --- | --- | --- |
| Yes | 92.86% | 65 |
| No | 7.14% | 5 |
| TOTAL |  | 70 |

Q22 Is your blood pressure well-controlled?

Answered: 70 Skipped: 1

| **ANSWER CHOICES** | **RESPONSES** |  |
| --- | --- | --- |
| Yes | 90.00% | 63 |
| No | 5.71% | 4 |
| I don't know | 4.29% | 3 |
| TOTAL |  | 70 |

Q23 Do you smoke?

Answered: 70 Skipped: 1

| **ANSWER CHOICES** | **RESPONSES** |  |
| --- | --- | --- |
| Yes | 0.00% | 0 |
| No | 100.00% | 70 |
| TOTAL |  | 70 |

Q24 Are you doing any physical activity?

Answered: 70 Skipped: 1

| **ANSWER CHOICES** | **RESPONSES** |  |
| --- | --- | --- |
| Yes. Intense regular exercise | 0.00% | 0 |
| Yes. Moderate regular physical activity | 41.43% | 29 |
| Yes. I don't exercise but I have an active lifestyle | 31.43% | 22 |
| No | 27.14% | 19 |
| TOTAL |  | 70 |

Q25 Did members of your family attend a Genetics appointment?

Answered: 70 Skipped: 1

| **ANSWER CHOICES** | **RESPONSES** |  |
| --- | --- | --- |
| Yes | 30.00% | 21 |
| No | 67.14% | 47 |
| I don't know | 2.86% | 2 |
| I prefer not to say | 0.00% | 0 |
| TOTAL |  | 70 |

Q26 If your family DID NOT attend a Genetics appointment, why was this?

Answered: 53 Skipped: 18

| **ANSWER CHOICES** | **RESPONSES** |  |
| --- | --- | --- |
| We were not offered one | 49.06% | 26 |
| We were told it wasn't necessary | 18.87% | 10 |
| It was offered but we chose not to attend | 1.89% | 1 |
| We have not yet attended but are planning to | 1.89% | 1 |
| Other (please specify) | 28.30% | 15 |
| TOTAL |  | 53 |

Q27 Did you or any of your relatives have a genetic test result where something was found (a positive test) after your aortic dissection?

Answered: 70 Skipped: 1

| **ANSWER CHOICES** | **RESPONSES** |  |
| --- | --- | --- |
| Yes | 14.29% | 10 |
| No | 85.71% | 60 |
| Prefer not to say | 0.00% | 0 |
| TOTAL |  | 70 |

Q28 Was there another case of aortic dissection in your family after yours?

Answered: 70 Skipped: 1

| **ANSWER CHOICES** | **RESPONSES** |  |
| --- | --- | --- |
| Yes | 5.71% | 4 |
| No | 92.86% | 65 |
| Prefer not to say | 1.43% | 1 |
| TOTAL |  | 70 |

Q29 After your aortic dissection, was another member of your family diagnosed with an aortic disease?

Answered: 70 Skipped: 1

| **ANSWER CHOICES** | **RESPONSES** |  |
| --- | --- | --- |
| Yes | 14.29% | 10 |
| No | 85.71% | 60 |
| Prefer not to say | 0.00% | 0 |
| TOTAL |  | 70 |

Q30 Sometimes, discussing genetic risks and performing screening can expose families to psychological stress. If you received genetic

counselling/testing, do you think that it created a psychological burden, such as increasing your level of anxiety and/or depression?

Answered: 68 Skipped: 3

| **ANSWER CHOICES** | **RESPONSES** |  |
| --- | --- | --- |
| Yes | 16.18% | 11 |
| No | 33.82% | 23 |
| I didn't have genetic testing/counselling | 50.00% | 34 |
| I prefer not to say | 0.00% | 0 |
| TOTAL |  | 68 |

Q31 Thinking about genetic testing, how would you rank the anxiety caused by each of the following factors? (1=lowest anxiety, 5=highest anxiety)

Answered: 67 Skipped: 4

|  | **1** | **2** | **3** | **4** | **5** | **6** | **7** | **TOTAL** | **SCORE** |
| --- | --- | --- | --- | --- | --- | --- | --- | --- | --- |
| The test results may be wrong and show a problem, even if there is no disease | 29.82%  17 | 26.32%  15 | 22.81%  13 | 8.77%  5 | 12.28%  7 | 0.00%  0 | 0.00%  0 | 57 | 5.53 |
| The test results may be wrong and not show a problem, even if there is disease | 16.36%  9 | 34.55%  19 | 16.36%  9 | 25.45%  14 | 7.27%  4 | 0.00%  0 | 0.00%  0 | 55 | 5.27 |
| The test results could be inconclusive | 25.93%  14 | 20.37%  11 | 35.19%  19 | 12.96%  7 | 5.56%  3 | 0.00%  0 | 0.00%  0 | 54 | 5.48 |
| The results could affect someone's insurance status | 0.00%  0 | 0.00%  0 | 0.00%  0 | 0.00%  0 | 0.00%  0 | 0.00%  0 | 0.00%  0 | 0 | 0.00 |
| The results could affect someone's career | 0.00%  0 | 0.00%  0 | 0.00%  0 | 0.00%  0 | 0.00%  0 | 0.00%  0 | 0.00%  0 | 0 | 0.00 |
| There could be no clear treatment plan as a consequence of the test results | 9.26%  5 | 9.26%  5 | 20.37%  11 | 27.78%  15 | 33.33%  18 | 0.00%  0 | 0.00%  0 | 54 | 4.33 |
| The results could require a participant to undergo many years of medical tests | 24.59%  15 | 13.11%  8 | 9.84%  6 | 22.95%  14 | 29.51%  18 | 0.00%  0 | 0.00%  0 | 61 | 4.80 |

Q32 Are you concerned about the potential implications of a genetic test result on your insurance status?

Answered: 69 Skipped: 2

| **ANSWER CHOICES** | **RESPONSES** |  |
| --- | --- | --- |
| Yes | 26.09% | 18 |
| No | 72.46% | 50 |
| Prefer not to say. | 1.45% | 1 |
| TOTAL |  | 69 |

Q33 Are you concerned about the potential implications of a genetic test result on your employment or career?

Answered: 70 Skipped: 1

| **ANSWER CHOICES** | **RESPONSES** |  |
| --- | --- | --- |
| Yes | 11.43% | 8 |
| No | 88.57% | 62 |
| Prefer not to say. | 0.00% | 0 |
| TOTAL |  | 70 |

Q34 In your experience, did you and your family receive adequate psychological support during follow-up after your aortic dissection?

Answered: 70 Skipped: 1

| **ANSWER CHOICES** | **RESPONSES** |  |
| --- | --- | --- |
| Yes | 10.00% | 7 |
| No | 74.29% | 52 |
| It varied | 14.29% | 10 |
| Prefer not to say | 1.43% | 1 |
| TOTAL |  | 70 |

Q35 When is the best time to approach patients and their families to talk to them about taking part in a trial of family screening?

Answered: 65 Skipped: 6

| **ANSWER CHOICES** | **RESPONSES** |  |
| --- | --- | --- |
| Before any surgery, if possible | 12.31% | 8 |
| During the hospital stay | 27.69% | 18 |
| During the follow-up, after discharge from the hospital | 60.00% | 39 |
| TOTAL |  | 65 |

Q36 Who should talk to patients and their families about taking part in a screening research project?

Answered: 63 Skipped: 8

| **ANSWER CHOICES** | **RESPONSES** |  |
| --- | --- | --- |
| Aortic Nurse | 34.92% | 22 |
| Aortic Surgeon | 22.22% | 14 |
| Cardiologist | 9.52% | 6 |
| Clinical Geneticist | 33.33% | 21 |
| TOTAL |  | 63 |

Q37 How many of your relatives, approximately, do you think would be willing to take part in a trial of screening for their risk of aortic disease?

Answered: 62 Skipped: 9

| **ANSWER CHOICES** | **RESPONSES** |  |
| --- | --- | --- |
| First Degree Relatives (parents, siblings or children): | 98.39% | 61 |
| Second Degree Relatives (uncles, aunts, nephews, nieces, grandparents, grandchildren): | ^91.94%^ | 57 |

Q38 In your opinion, what are the best ways to measure the success of a screening program for relatives of those who have aortic disease?

[Please rank: 8=the best, 1=the worst)

Answered: 63 Skipped: 8

|  | **1** | **2** | **3** | **4** | **5** | **6** | **7** | **8** | **TOTAL** | **SCORE** |
| --- | --- | --- | --- | --- | --- | --- | --- | --- | --- | --- |
| Reduction in deaths | 12.28%  7 | 7.02%  4 | 1.75%  1 | 8.77%  5 | 7.02%  4 | 5.26%  3 | 21.05%  12 | 36.84%  21 | 57 | 3.25 |
| Reduction in aortic dissections | 7.02%  4 | 10.53%  6 | 7.02%  4 | 0.00%  0 | 5.26%  3 | 8.77%  5 | 33.33%  19 | 28.07%  16 | 57 | 3.14 |
| Earlier intervention on aortic aneurysms | 5.45%  3 | 7.27%  4 | 12.73%  7 | 10.91%  6 | 12.73%  7 | 27.27%  15 | 9.09%  5 | 14.55%  8 | 55 | 3.91 |
| More families accessing genetic testing | 5.17%  3 | 12.07%  7 | 18.97%  11 | 24.14%  14 | 13.79%  8 | 12.07%  7 | 10.34%  6 | 3.45%  2 | 58 | 4.76 |
| More families accessing imaging surveillance | 8.62%  5 | 10.34%  6 | 20.69%  12 | 20.69%  12 | 18.97%  11 | 8.62%  5 | 6.90%  4 | 5.17%  3 | 58 | 4.90 |
| More effective control of blood pressure | 8.77%  5 | 15.79%  9 | 10.53%  6 | 14.04%  8 | 21.05%  12 | 22.81%  13 | 3.51%  2 | 3.51%  2 | 57 | 4.77 |
| Reduction in anxiety or depression | 18.64%  11 | 27.12%  16 | 18.64%  11 | 8.47%  5 | 10.17%  6 | 5.08%  3 | 6.78%  4 | 5.08%  3 | 59 | 5.68 |
| No reduction in quality of life as a result of screening | 31.67%  19 | 8.33%  5 | 6.67%  4 | 13.33%  8 | 10.00%  6 | 11.67%  7 | 8.33%  5 | 10.00%  6 | 60 | 5.20 |

Q39 If you participated in a trial of family screening, in addition to the information that would be collected as part of your standard care, would you be willing to send other details to clinicians and researchers, using a mobile phone app?

Answered: 63 Skipped: 8

| **ANSWER CHOICES** | **RESPONSES** |  |
| --- | --- | --- |
| Yes | 96.83% | 61 |
| No | 3.17% | 2 |
| TOTAL |  | 63 |

Q40 If you participated in a clinical trial of screening, how much time would you be willing to spend per week submitting data on a mobile phone app, to help the research? (minutes per week)

Answered: 63 Skipped: 8

| **#** | **RESPONSES** |  |
| --- | --- | --- |
| 1 | 2 hrs |  |
| 2 | 30 |  |
| 3 | 70 mins per week |  |
| 4 | 60 |  |
| 5 | 15 |  |
| 6 | 1 hour |  |
| 7 | 15 |  |
| 8 | 30 |  |
| 9 | 120 |  |
| 10 | 20 |  |
| 11 | 20 minutes |  |
| 12 | 60 |  |
| 13 | 60 |  |
| 14 | 60-70 (10mns a day) |  |
| 15 | 30 |  |
| 16 | 10 |  |
| 17 | 15 |  |
| 18 | 20 |  |
| 19 | 60 |  |
| 20 | 30 |  |
| 21 | 30 |  |
| 22 | 30 |  |
| 23 | 60 |  |
| 24 | 20 |  |
| 25 | 180 minutes |  |
| 26 | 30 |  |
| 27 | 60 |  |
| 28 | 60 |  |
| 29 | 180 |  |
| 30 | 30 |  |
| 31 | 60 |  |
| 32 | 15 |  |
| 33 | 60 |  |
| 34 | 60 |  |
| 35 | 10 |  |
| 36 | 180 |  |
| 37 | 360 |  |
| 38 | 60 |  |
| 39 | 30 |  |
| 40 | 45 |  |
| 41 | 60 |  |
| 42 | 60 |  |
| 43 | 60 |  |
| 44 | 60 |  |
| 45 | 10 |  |
| 46 | 60 |  |
| 47 | 10 |  |
| 48 | 0 |  |
| 49 | Any |  |
| 50 | any |  |
| 51 | 60 |  |
| 52 | 30 |  |
| 53 | 30 |  |
| 54 | Whatever is necessary |  |
| 55 | 15 |  |
| 56 | 30 |  |
| 57 | 30 |  |
| 58 | 30 |  |
| 59 | 30 |  |
| 60 | 30 |  |
| 61 | 90 |  |
| 62 | 30 |  |
| 63 | 30 |  |

Q41 How far you would be willing to travel to undergo screening? (miles from home)

Answered: 63 Skipped: 8

| **#** | **RESPONSES** |
| --- | --- |
| 1 | 10 |
| 2 | 100 |
| 3 | Unable to travel |
| 4 | 50 |
| 5 | 30 |
| 6 | An hour |
| 7 | 50 |
| 8 | 50 |
| 9 | 40 |
| 10 | Not sure |
| 11 | 45 miles |
| 12 | 30 |
| 13 | 80 |
| 14 | 10-20 |
| 15 | 60 |
| 16 | 70 |
| 17 | 25 |
| 18 | 30 |
| 19 | 30 |
| 20 | 10 |
| 21 | 15 cost of fuel/ transport issues |
| 22 | 30 |
| 23 | 50 |
| 24 | 25 |
| 25 | 10 |
| 26 | 30 |
| 27 | 15 |
| 28 | 20 |
| 29 | 25 |
| 30 | 5 |
| 31 | 140 |
| 32 | Who's paying? 40 miles. |
| 33 | 10 |
| 34 | 50 |
| 35 | 120 |
| 36 | 30 |
| 37 | 200 |
| 38 | 20 |
| 39 | 20 |
| 40 | 20 |
| 41 | 100 |
| 42 | 30 |
| 43 | 50 |
| 44 | 25 |
| 45 | 50 |
| 46 | 100 |
| 47 | 20 |
| 48 | 0 |
| 49 | Any |
| 50 | 100 |
| 51 | 75 |
| 52 | 25 |
| 53 | 100 |
| 54 | I would have to travel |
| 55 | 10 |
| 56 | 20 |
| 57 | 25 |
| 58 | 15 |
| 59 | 100 |
| 60 | 100 |
| 61 | 20 |
| 62 | 10 |
| 63 | 100 |

Q42 Around half of the people who are screened for a genetic cause of aortic disease have an inconclusive result, one which cannot yet be interpreted with our current knowledge. This is known as a 'Variant of Uncertain Significance' or 'VUS'. Although no changes to the care of the patient and family follow from such a test, the result would need to be reassessed in future, as our knowledge grows. If the result of your family's genetic testing was inconclusive, how distressed do you think you would you feel, on a scale of 1-10? (Move the slider: 0=not at all distressed, 100=very distressed).

Answered: 60 Skipped: 11

|  | **AVERAGE NUMBER** |  |  |  | **RESPONSES** |  |
| --- | --- | --- | --- | --- | --- | --- |
|  |  | 39 |  |  |  | 60 |
|  |  |  |  |  |  |  |

Q43 Is your or your family’s care managed by a clinic that specialises in screening, diagnosing, counselling and treating patients with inherited cardiac conditions (sometimes known as an Inherited Cardiac Conditions or ICC centre)?

Answered: 63 Skipped: 8

| **ANSWER CHOICES** | **RESPONSES** |  |
| --- | --- | --- |
| Yes | 17.46% | 11 |
| No | 55.56% | 35 |
| I don't know | 26.98% | 17 |
| TOTAL |  | 63 |

Q44 Which of these people should have access to the Decision Support

Tool, if it was to include your individual health data? (select all that apply)

Answered: 63 Skipped: 8

| **ANSWER CHOICES** | **RESPONSES** |  |  | |  |
| --- | --- | --- | --- | --- | --- |
| Me, as the patient | 90.48% |  |  | | 57 |
| My relatives | 42.86% |  |  | | 27 |
| Surgeons | 82.54% |  |  | | 52 |
| Cardiologists | 90.48% |  |  | | 57 |
| Clinical Geneticists | 80.95% |  |  | | 51 |
| Aortic Nurses | 74.60% |  |  | | 47 |
| GP | 87.30% |  |  | | 55 |
| Other (please specify) | 4.76% |  |  | | 3 |
|  |  |  |  | |  |
|  |  | | |  | |

Q45 If you & your family used a Decision Support Tool, would you be concerned about the possibility that other family members may learn of your test results indirectly through the Decision Support Tool?

Answered: 62 Skipped: 9

| **ANSWER CHOICES** | **RESPONSES** |  |
| --- | --- | --- |
| Yes | 17.74% | 11 |
| No | 82.26% | 51 |
| TOTAL |  | 62 |

Q46 Which of the following was your GP able to provide you with information about (tick all that apply):

Answered: 63 Skipped: 8

| **ANSWER CHOICES** | **RESPONSES** |  |
| --- | --- | --- |
| Levels of physical activity that you can/should have | 6.35% | 4 |
| General lifestyle advice | 17.46% | 11 |
| Medications that you should take. | 36.51% | 23 |
| Tests that you should undergo | 6.35% | 4 |
| Implications for your family | 6.35% | 4 |
| None of the above | 60.32% | 38 |
| Total Respondents: 63 |  |  |

Q47 In general, do you feel that you were provided with adequate information and included in decision-making about your care, as an aortic patient?

Answered: 63 Skipped: 8

| **ANSWER CHOICES** | **RESPONSES** |  |
| --- | --- | --- |
| Yes | 30.16% | 19 |
| No | 65.08% | 41 |
| I prefer not to say | 4.76% | 3 |
| TOTAL |  | 63 |

Q48 Which of the following medical specialists have been involved in your or your family's care for your aortic disease? (tick all that apply)

Answered: 63 Skipped: 8

| **ANSWER CHOICES** | **RESPONSES** |  |  |
| --- | --- | --- | --- |
| General Practitioner | 73.02% |  | 46 |
| Cardiac Surgeons | 76.19% |  | 48 |
| Vascular Surgeons | 57.14% |  | 36 |
| Cardiologists | 53.97% |  | 34 |
| Clinical Geneticists | 36.51% |  | 23 |
| Radiologists | 55.56% |  | 35 |
| Psychologists | 6.35% |  | 4 |
| Aortic Nurses | 34.92% |  | 22 |
| Other (specify: ____) | 6.35% |  | 4 |
| Total Respondents: 63 |  |  |  |

Q49 Was there effective communication between the different specialist groups involved in your care?

Answered: 63 Skipped: 8

| **ANSWER CHOICES** | **RESPONSES** |  |
| --- | --- | --- |
| Yes | 22.22% | 14 |
| No | 28.57% | 18 |
| It varies | 49.21% | 31 |
| Prefer not to say | 0.00% | 0 |
| TOTAL |  | 63 |

Q50 Follow-up of aortic patients might require the involvement of different specialist clinical groups across different hospitals and NHS Trusts. Was this ever a source of problems, in your personal experience?

Answered: 63 Skipped: 8

| **ANSWER CHOICES** | **RESPONSES** |  |
| --- | --- | --- |
| Yes | 53.97% | 34 |
| No | 39.68% | 25 |
| Prefer not to say | 6.35% | 4 |
| TOTAL |  | 63 |

Q51 How familiar are you with using digital devices such as mobile phones, iPads or computers)?

Answered: 63 Skipped: 8

|  | **VERY FAMILIAR** | **SOMEWHAT FAMILIAR** | **NOT FAMILIAR** | **TOTAL** | **WEIGHTED AVERAGE** |
| --- | --- | --- | --- | --- | --- |
| Mobile Phone | 84.13%  53 | 15.87%  10 | 0.00%  0 | 63 | 1.16 |
| Tablet (e.g. iPad) | 68.85%  42 | 27.87%  17 | 3.28%  2 | 61 | 1.34 |
| Computer | 75.81%  47 | 20.97%  13 | 3.23%  2 | 62 | 1.27 |

Q52 Where do you most often go for medical information in your everyday life?

Answered: 63 Skipped: 8

| **ANSWER CHOICES** | **RESPONSES** |  |  |
| --- | --- | --- | --- |
| Consult with clinicians. | 33.33% |  | 21 |
| Ask other patients. | 7.94% |  | 5 |
| The Internet. | 46.03% |  | 29 |
| The Media. | 1.59% |  | 1 |
| Other (please specify) | 11.11% |  | 7 |
| TOTAL |  |  | 63 |
|  |  |  | |

Q53 Which of the following information do you think patients and their families would benefit from knowing when getting involved in screening?

(tick all that apply)

Answered: 63 Skipped: 8

| **ANSWER CHOICES** | **RESPONSES** | |
| --- | --- | --- |
| Information about the disease for the participant, their family and their primary health care providers (e.g. GP) | 95.24% | 60 |
| Information about the screening tests to be performed | 85.71% | 54 |
| The implications of a positive genetic test (where something is found) | 82.54% | 52 |
| The implications of a negative genetic test (where nothing is found) | 58.73% | 37 |
| The implications of an uncertain genetic test | 66.67% | 42 |
| What happens when there is no clear diagnosis | 61.90% | 39 |
| When and how often imaging surveillance will occur | 73.02% | 46 |
| Information about lifestyle advice and medication that might be given for prevention | 80.95% | 51 |
| Reasons for any proposed treatment that might be offered | 77.78% | 49 |
| Information about potential future surgery | 80.95% | 51 |
| Total Respondents: 63 |  |  |

Q54 How would you prefer to receive the information required for shared decision making about screening?

Answered: 63 Skipped: 8

| **ANSWER CHOICES** | **RESPONSES** |  |  |
| --- | --- | --- | --- |
| A booklet | 38.10% |  | 24 |
| A mobile phone app | 26.98% |  | 17 |
| A website | 30.16% |  | 19 |
| A video | 0.00% |  | 0 |
| Other (please specify) | 4.76% |  | 3 |
| TOTAL |  |  | 63 |
|  |  |  | |

Q55 Which of the following information is most important for a family like yours affected by aortic dissection? [Rate each item]

Answered: 63 Skipped: 8

|  | **UNIMPORTANT** | **NICE**  **TO**  **HAVE** | **IMPORTANT** | **VERY**  **IMPORTANT** | **ESSENTIAL** | **TOTAL** | **WEIGHTED AVERAGE** |
| --- | --- | --- | --- | --- | --- | --- | --- |
| Understanding the possible diagnosis | 0.00%  0 | 3.17%  2 | 22.22%  14 | 31.75%  20 | 42.86%  27 | 63 | 4.14 |
| Understanding the possible results of imaging tests | 0.00%  0 | 1.59%  1 | 28.57%  18 | 38.10%  24 | 31.75%  20 | 63 | 4.00 |
| Understanding the possible results of a genetic test | 1.59%  1 | 3.17%  2 | 33.33%  21 | 38.10%  24 | 23.81%  15 | 63 | 3.79 |
| What does a positive imaging test mean for the patient? | 0.00%  0 | 3.17%  2 | 17.46%  11 | 42.86%  27 | 36.51%  23 | 63 | 4.13 |
| What does a negative imaging test mean for the patient? | 0.00%  0 | 6.35%  4 | 38.10%  24 | 36.51%  23 | 19.05%  12 | 63 | 3.68 |
| How will the results of the imaging test inform future care? | 0.00%  0 | 1.64%  1 | 26.23%  16 | 39.34%  24 | 32.79%  20 | 61 | 4.03 |
| What does a positive genetic test mean for the patient? | 0.00%  0 | 3.23%  2 | 14.52%  9 | 37.10%  23 | 45.16%  28 | 62 | 4.24 |
| What does a negative genetic test mean for the patient? | 1.61%  1 | 11.29%  7 | 40.32%  25 | 30.65%  19 | 16.13%  10 | 62 | 3.48 |
| How will the results of the genetic test inform future care? | 0.00%  0 | 1.61%  1 | 30.65%  19 | 38.71%  24 | 29.03%  18 | 62 | 3.95 |
| How frequent will tests be? | 3.17%  2 | 7.94%  5 | 50.79%  32 | 15.87%  10 | 22.22%  14 | 63 | 3.46 |
| What are the treatment options? | 0.00%  0 | 3.23%  2 | 30.65%  19 | 30.65%  19 | 35.48%  22 | 62 | 3.98 |
| What are the consequences of treatment? | 0.00%  0 | 1.59%  1 | 25.40%  16 | 31.75%  20 | 41.27%  26 | 63 | 4.13 |
| How do we know if treatment is working/will work? | 0.00%  0 | 1.59%  1 | 22.22%  14 | 36.51%  23 | 39.68%  25 | 63 | 4.14 |
| If the results of screening are inconclusive, what happens next? | 0.00%  0 | 6.35%  4 | 41.27%  26 | 30.16%  19 | 22.22%  14 | 63 | 3.68 |
| Other: … | 0.00%  0 | 0.00%  0 | 0.00%  0 | 0.00%  0 | 0.00%  0 | 0 | 0.00 |

Q56 If we require further patient input, would you be willing to be contacted in future with requests to help our research activities to develop a Decision Support Tool?

Answered: 62 Skipped: 9

| **ANSWER CHOICES** | **RESPONSES** |  |
| --- | --- | --- |
| Yes | 87.10% | 54 |
| No | 12.90% | 8 |
| TOTAL |  | 62 |

Q57 If you were to become involved in helping us with this research, how would you prefer to do this? (select your 1st preference)

Answered: 58 Skipped: 13

| **ANSWER CHOICES** | **RESPONSES** |  |
| --- | --- | --- |
| Face-to-face meeting | 17.24% | 10 |
| Telephone call | 18.97% | 11 |
| Video call | 25.86% | 15 |
| E-mail | 37.93% | 22 |
| TOTAL |  | 58 |

*Family members*

Q1 How are you related to someone who had an Aortic Dissection?

Answered: 173 Skipped: 0

| **ANSWER CHOICES** | **RESPONSES** | |
| --- | --- | --- |
| I am a first-degree blood relative (parent, child, brother or sister) of someone who had an Aortic Dissection | 89.02% | 154 |
| I am a second-degree blood relative (grandparent, grandchild, uncle, aunt, nephew, niece) of someone who had an  Aortic Dissection | 9.25% | 16 |
| Other (please specify) | 1.73% | 3 |
| TOTAL |  | 173 |

Q2 In which region of the UK do you live?

Answered: 169 Skipped: 4

| **ANSWER CHOICES** | **RESPONSES** |  |
| --- | --- | --- |
| East | 4.73% | 8 |
| East Midlands | 10.06% | 17 |
| London | 10.65% | 18 |
| North East | 3.55% | 6 |
| North West | 11.24% | 19 |
| Northern Ireland | 2.37% | 4 |
| Scotland | 4.14% | 7 |
| South East | 20.71% | 35 |
| South West | 14.20% | 24 |
| Cymru/Wales | 4.14% | 7 |
| West Midlands | 6.51% | 11 |
| Yorkshire And The Humber | 7.69% | 13 |
| TOTAL |  | 169 |

Q3 What is your gender?

Answered: 173 Skipped: 0

| **ANSWER CHOICES** | **RESPONSES** |  |
| --- | --- | --- |
| Female | 59.54% | 103 |
| Male | 39.31% | 68 |
| Prefer not to say | 1.16% | 2 |
| TOTAL |  | 173 |

Q4 What is your current age (in years)?

Answered: 171 Skipped: 2

| **Number** | 171 |
| --- | --- |
| **Age** (Years) **Mean** (IQR) | 42.5 (31.3 - 57.6) |

Q5 Which ethnic group do you belong to?

Answered: 173 Skipped: 0

| **ANSWER CHOICES** | **RESPONSES** |  |
| --- | --- | --- |
| English, Welsh, Scottish, Northern Irish or British | 90.75% | 157 |
| Irish | 3.47% | 6 |
| Gypsy or Irish Traveller | 0.00% | 0 |
| Any other white background | 1.16% | 2 |
| White and Black Caribbean | 0.00% | 0 |
| White and Black African | 0.00% | 0 |
| White and Asian | 0.58% | 1 |
| Any other mixed or multiple ethnic background | 2.31% | 4 |
| Indian | 0.58% | 1 |
| Pakistani | 0.00% | 0 |
| Bangladeshi | 0.00% | 0 |
| Chinese | 0.00% | 0 |
| Any other Asian background | 0.00% | 0 |
| African | 0.00% | 0 |
| Caribbean | 0.58% | 1 |
| Any other Black, African or Caribbean background | 0.00% | 0 |
| Any other ethnic group | 0.00% | 0 |
| Other (please specify) | 0.58% | 1 |
| Total Respondents: 173 |  |  |
| **#** | **OTHER (PLEASE SPECIFY)** | **DATE** |
| 1 | Cornish | 2/3/2023 9:24 AM |

Q6 Had you ever heard about aortic dissection before your relative(s) had one?

Answered: 165 Skipped: 8

| **ANSWER CHOICES** | **RESPONSES** |  |
| --- | --- | --- |
| Yes, from someone close to me | 5.45% | 9 |
| Yes, from healthcare professionals | 4.24% | 7 |
| Yes, from other sources | 9.09% | 15 |
| No, I had never heard about it | 81.21% | 134 |
| TOTAL |  | 165 |

Q7 Had you ever received specialist care for a cardiovascular condition (diseases of your heart and/or arteries)?

Answered: 163 Skipped: 10

| **ANSWER CHOICES** | **RESPONSES** |  |
| --- | --- | --- |
| Yes, I previously saw a specialist and I had a planned follow-up | 6.75% | 11 |
| Yes, I previously saw a specialist appointment, but I had no planned follow-up | 3.68% | 6 |
| No, I never received any specialist care for a cardiovascular condition | 89.57% | 146 |
| TOTAL |  | 163 |

Q8 Was your blood pressure regularly monitored?

Answered: 164 Skipped: 9

| **ANSWER CHOICES** | **RESPONSES** |  |
| --- | --- | --- |
| Yes, I monitored it myself at home | 8.54% | 14 |
| Yes, my GP monitored it | 12.80% | 21 |
| No | 78.66% | 129 |
| TOTAL |  | 164 |

Q9 Did you smoke?

Answered: 164 Skipped: 9

| **ANSWER CHOICES** | **RESPONSES** |  |
| --- | --- | --- |
| I never smoked | 68.29% | 112 |
| I smoked in the past, but I had stopped | 22.56% | 37 |
| I smoked | 9.15% | 15 |
| TOTAL |  | 164 |

Q10 What was your level of physical activity?

Answered: 164 Skipped: 9

| **ANSWER CHOICES** | **RESPONSES** |  |
| --- | --- | --- |
| Intense physical activity, for example, weightlifting in the gym | 14.63% | 24 |
| Fitness exercise and generally an active lifestyle | 71.95% | 118 |
| Sedentary lifestyle, for example, never walking more than 200 yards regularly | 13.41% | 22 |
| TOTAL |  | 164 |

Q11 Had you been diagnosed with any aortic syndrome such as Marfan, Ehlers-Danlos, Loeys-Dietz, or Osteogenesis Imperfecta (brittle bones)?

Answered: 164 Skipped: 9

| **ANSWER CHOICES** | **RESPONSES** |  |
| --- | --- | --- |
| Yes | 3.05% | 5 |
| No | 96.95% | 159 |
| TOTAL |  | 164 |

Q12 Did you ever have a Genetics appointment?

Answered: 163 Skipped: 10

| **ANSWER CHOICES** | **RESPONSES** |  |
| --- | --- | --- |
| Yes | 9.20% | 15 |
| No | 87.73% | 143 |
| I don’t know | 2.45% | 4 |
| I prefer not to say | 0.61% | 1 |
| TOTAL |  | 163 |

Q13 In the past, was there ever a sudden death among your blood relatives?

Answered: 160 Skipped: 13

| **ANSWER CHOICES** | **RESPONSES** |  |
| --- | --- | --- |
| Yes | 48.13% | 77 |
| No | 42.50% | 68 |
| I don’t know | 9.38% | 15 |
| I prefer not to say | 0.00% | 0 |
| TOTAL |  | 160 |

Q14 In the past, were there any known aortic aneurysms among your blood relatives, or did anyone previously require treatment for their aorta?

Answered: 160 Skipped: 13

| **ANSWER CHOICES** | **RESPONSES** |  |
| --- | --- | --- |
| Yes | 34.38% | 55 |
| No | 45.00% | 72 |
| I don’t know | 20.63% | 33 |
| I prefer not to say | 0.00% | 0 |
| TOTAL |  | 160 |

Q15 In the past, was there ever an aortic dissection among your blood relatives?

Answered: 160 Skipped: 13

| **ANSWER CHOICES** | **RESPONSES** |  |
| --- | --- | --- |
| Yes | 36.25% | 58 |
| No | 50.00% | 80 |
| I don’t know | 13.75% | 22 |
| I prefer not to say | 0.00% | 0 |
| TOTAL |  | 160 |

Q16 Were any of your blood relatives ever diagnosed with any syndrome, such as Marfan, Ehlers-Danlos, Loeys-Dietz, or Osteogenesis Imperfecta (brittle bones)?

Answered: 160 Skipped: 13

| **ANSWER CHOICES** | **RESPONSES** |  |
| --- | --- | --- |
| Yes | 7.50% | 12 |
| No | 76.25% | 122 |
| I don't know | 16.25% | 26 |
| I prefer not to say | 0.00% | 0 |
| TOTAL |  | 160 |

Q17 Did any of your blood relatives receive specialist care for cardiovascular conditions (diseases of the heart and arteries)?

Answered: 159 Skipped: 14

| **ANSWER CHOICES** | **RESPONSES** |  |
| --- | --- | --- |
| Yes | 42.14% | 67 |
| No | 44.03% | 70 |
| I don't know | 13.84% | 22 |
| I prefer not to say | 0.00% | 0 |
| TOTAL |  | 159 |

Q18 Did a healthcare professional ever tell you about the possible implications for your family due to the genetic nature of aortic dissection?

| **ANSWER CHOICES** | **RESPONSES** |  |
| --- | --- | --- |
| Yes, before my relative(s) had an aortic dissection | 0.65% | 1 |
| Yes, when a relative was in hospital for aortic dissection | 5.19% | 8 |
| Yes, after a relative had an aortic dissection | 35.06% | 54 |
| No, I learned of it through the patient charity | 8.44% | 13 |
| No, I learned of it through the internet or social media | 7.79% | 12 |
| No, I learned it from other sources | 14.29% | 22 |
| No, I was not aware of any such implications | 28.57% | 44 |
| TOTAL |  | 154 |

Answered: 154 Skipped: 19

Q19 Was any form of counselling or support arranged for you after your relative(s) had an aortic dissection?

Answered: 153 Skipped: 20

| **ANSWER CHOICES** | **RESPONSES** |  |
| --- | --- | --- |
| Yes, and It helped | 3.92% | 6 |
| Yes, but it didn't help | 1.31% | 2 |
| It was offered, but I didn't take it up | 2.61% | 4 |
| No, but I think it would have helped | 66.67% | 102 |
| No, and I don't think it would have helped | 25.49% | 39 |
| TOTAL |  | 153 |

Q20 Did you have genetic testing?

Answered: 155 Skipped: 18

| **ANSWER CHOICES** | **RESPONSES** |  |
| --- | --- | --- |
| Yes | 20.65% | 32 |
| No, but I was offered it | 1.94% | 3 |
| No, I wasn't offered it | 77.42% | 120 |
| TOTAL |  | 155 |

Q21 Are you taking medications for your blood pressure?

Answered: 155 Skipped: 18

| **ANSWER CHOICES** | **RESPONSES** |  |
| --- | --- | --- |
| Yes | 21.29% | 33 |
| No | 78.71% | 122 |
| TOTAL |  | 155 |

Q22 Is your blood pressure well-controlled?

Answered: 154 Skipped: 19

| **ANSWER CHOICES** | **RESPONSES** |  |
| --- | --- | --- |
| Yes | 57.79% | 89 |
| No | 6.49% | 10 |
| I don't know | 35.71% | 55 |
| TOTAL |  | 154 |

Q23 Do you smoke?

Answered: 155 Skipped: 18

| **ANSWER CHOICES** | **RESPONSES** |  |
| --- | --- | --- |
| Yes | 9.03% | 14 |
| No | 90.97% | 141 |
| TOTAL |  | 155 |

Q24 Are you doing any physical activity?

Answered: 154 Skipped: 19

| **ANSWER CHOICES** | **RESPONSES** |  |
| --- | --- | --- |
| Yes. Intense regular exercise | 17.53% | 27 |
| Yes. Moderate regular physical activity | 51.95% | 80 |
| Yes. I don't exercise but I have an active lifestyle | 20.78% | 32 |
| No | 9.74% | 15 |
| TOTAL |  | 154 |

Q25 Did you or your family attend a Genetics appointment after your relative(s)' aortic dissection?

Answered: 151 Skipped: 22

| **ANSWER CHOICES** | **RESPONSES** |  |
| --- | --- | --- |
| Yes | 29.80% | 45 |
| No | 67.55% | 102 |
| I don't know | 2.65% | 4 |
| I prefer not to say | 0.00% | 0 |
| TOTAL |  | 151 |

Q26 If you DID NOT attend a Genetics appointment, why was this?

Answered: 123 Skipped: 50

| **ANSWER CHOICES** | **RESPONSES** |  |
| --- | --- | --- |
| We were not offered one | 73.98% | 91 |
| We were told it wasn't necessary | 7.32% | 9 |
| It was offered but we chose not to attend | 0.00% | 0 |
| We have not yet attended but are planning to | 1.63% | 2 |
| Other (please specify) | 17.07% | 21 |
| TOTAL |  | 123 |

Q27 Did you or any of your relatives have a genetic test result where something was found (a positive test) after your relative(s)' aortic dissection?

Answered: 147 Skipped: 26

| **ANSWER CHOICES** | **RESPONSES** |  |
| --- | --- | --- |
| Yes | 13.61% | 20 |
| No | 84.35% | 124 |
| Prefer not to say | 2.04% | 3 |
| TOTAL |  | 147 |

Q28 Did you have an imaging test, such as a CT or MRI scan, or an Echocardiogram (ultrasound scan of your heart & aorta)?

Answered: 150 Skipped: 23

| **ANSWER CHOICES** | **RESPONSES** |  |
| --- | --- | --- |
| Yes | 44.00% | 66 |
| No | 54.00% | 81 |
| I don't know | 2.00% | 3 |
| I prefer not to say | 0.00% | 0 |
| TOTAL |  | 150 |

Q29 Were you or any of your relatives diagnosed with an aortic aneurysm?

Answered: 149 Skipped: 24

| **ANSWER CHOICES** | **RESPONSES** |  |
| --- | --- | --- |
| Yes | 21.48% | 32 |
| No | 64.43% | 96 |
| I don't know | 14.09% | 21 |
| I prefer not to say | 0.00% | 0 |
| TOTAL |  | 149 |

Q30 Was there another case of aortic dissection in your family after your first relative who had an aortic dissection?

Answered: 150 Skipped: 23

| **ANSWER CHOICES** | **RESPONSES** |  |
| --- | --- | --- |
| Yes | 23.33% | 35 |
| No | 75.33% | 113 |
| Prefer not to say | 1.33% | 2 |
| TOTAL |  | 150 |

Q31 Is your family still under the care of a Geneticist?

Answered: 149 Skipped: 24

| **ANSWER CHOICES** | **RESPONSES** |  |
| --- | --- | --- |
| Yes | 18.12% | 27 |
| No | 80.54% | 120 |
| Prefer not to say | 1.34% | 2 |
| TOTAL |  | 149 |

Q32 Sometimes, discussing genetic risks and performing screening can expose families to psychological stress. If you received genetic

counselling/testing, do you think that it created a psychological burden, such as increasing your level of anxiety and/or depression?

Answered: 147 Skipped: 26

| **ANSWER CHOICES** | **RESPONSES** |  |
| --- | --- | --- |
| Yes | 10.88% | 16 |
| No | 18.37% | 27 |
| I didn't have genetic testing/counselling | 69.39% | 102 |
| I prefer not to say | 1.36% | 2 |
| TOTAL |  | 147 |

Q33 Thinking about genetic testing, how would you rank the anxiety caused by each of the following factors? (1=lowest anxiety, 5=highest anxiety)

Answered: 138 Skipped: 35

|  | **1** | **2** | **3** | **4** | **5** | **6** | **7** | **TOTAL** | **SCORE** |
| --- | --- | --- | --- | --- | --- | --- | --- | --- | --- |
| The test results may be wrong and show a problem, even if there is no disease | 34.82%  39 | 17.86%  20 | 18.75%  21 | 15.18%  17 | 13.39%  15 | 0.00%  0 | 0.00%  0 | 112 | 5.46 |
| The test results may be wrong and not show a problem, even if there is disease | 18.97%  22 | 28.45%  33 | 22.41%  26 | 18.10%  21 | 12.07%  14 | 0.00%  0 | 0.00%  0 | 116 | 5.24 |
| The test results could be inconclusive | 17.95%  21 | 23.08%  27 | 31.62%  37 | 13.68%  16 | 13.68%  16 | 0.00%  0 | 0.00%  0 | 117 | 5.18 |
| The results could affect someone's insurance status | 0.00%  0 | 0.00%  0 | 0.00%  0 | 0.00%  0 | 0.00%  0 | 0.00%  0 | 0.00%  0 | 0 | 0.00 |
| The results could affect someone's career | 0.00%  0 | 0.00%  0 | 0.00%  0 | 0.00%  0 | 0.00%  0 | 0.00%  0 | 0.00%  0 | 0 | 0.00 |
| There could be no clear treatment plan as a consequence of the test results | 12.07%  14 | 16.38%  19 | 17.24%  20 | 31.03%  36 | 23.28%  27 | 0.00%  0 | 0.00%  0 | 116 | 4.63 |
| The results could require a participant to undergo many years of medical tests | 21.21%  28 | 15.15%  20 | 14.39%  19 | 18.18%  24 | 31.06%  41 | 0.00%  0 | 0.00%  0 | 132 | 4.77 |

Q34 Are you concerned about the potential implications of a genetic test result on your insurance status?

Answered: 147 Skipped: 26

| **ANSWER CHOICES** | **RESPONSES** |  |
| --- | --- | --- |
| Yes | 29.93% | 44 |
| No | 68.03% | 100 |
| Prefer not to say. | 2.04% | 3 |
| TOTAL |  | 147 |

Q35 Are you concerned about the potential implications of a genetic test result on your employment or career?

Answered: 147 Skipped: 26

| **ANSWER CHOICES** | **RESPONSES** |  |
| --- | --- | --- |
| Yes | 17.69% | 26 |
| No | 80.95% | 119 |
| Prefer not to say. | 1.36% | 2 |
| TOTAL |  | 147 |

Q36 In your experience, did you and your family receive adequate psychological support after your relative's aortic dissection?

Answered: 147 Skipped: 26

| **ANSWER CHOICES** | **RESPONSES** |  |
| --- | --- | --- |
| Yes | 6.12% | 9 |
| No | 72.79% | 107 |
| It varied | 15.65% | 23 |
| Prefer not to say | 5.44% | 8 |
| TOTAL |  | 147 |

Q37 When is the best time to approach a surviving Aortic Dissection patient and their relatives to talk to them about taking part in a trial of family screening?

Answered: 130 Skipped: 43

| **ANSWER CHOICES** | **RESPONSES** |  |
| --- | --- | --- |
| Before any surgery, if possible | 17.69% | 23 |
| During the hospital stay, if possible | 24.62% | 32 |
| During the follow-up, after discharge from the hospital, if the patient survives | 57.69% | 75 |
| TOTAL |  | 130 |

Q38 When is the best time to approach the relatives of someone who has died as a result of Aortic Dissection to talk to them about taking part in a trial of family screening?

Answered: 131 Skipped: 42

| **ANSWER CHOICES** | **RESPONSES** |  |  |
| --- | --- | --- | --- |
| As soon as the death occurs | 25.95% |  | 34 |
| After the funeral | 64.12% |  | 84 |
| Never | 0.00% |  | 0 |
| Other (please specify) | 9.92% |  | 13 |
| TOTAL |  |  | 131 |

Q39 Who should talk to patients and their families about taking part in a screening research project?

Answered: 130 Skipped: 43

| **ANSWER CHOICES** | **RESPONSES** |  |
| --- | --- | --- |
| Aortic Nurse | 31.54% | 41 |
| Aortic Surgeon | 14.62% | 19 |
| Cardiologist | 20.00% | 26 |
| Clinical Geneticist | 33.85% | 44 |
| TOTAL |  | 130 |

Q40 How many of your relatives, approximately, do you think would be willing to take part in a trial of screening for their risk of aortic disease?

Answered: 122 Skipped: 51

| **ANSWER CHOICES** | **RESPONSES** |
| --- | --- |
| First Degree Relatives (parents, siblings or children): | 100.00% 122 |
| Second Degree Relatives (uncles, aunts, nephews, nieces, grandparents, grandchildren): | ^81.97%^  100 |

Q41 In your opinion, what are the best ways to measure the success of a screening program for relatives of those who have aortic disease?

[Please rank: 8=the best, 1=the worst)

Answered: 130 Skipped: 43

|  | **1** | **2** | **3** | **4** | **5** | **6** | **7** | **8** | **TOTAL** | **SCORE** |
| --- | --- | --- | --- | --- | --- | --- | --- | --- | --- | --- |
| Reduction in deaths | 24.58%  29 | 11.02%  13 | 5.08%  6 | 7.63%  9 | 3.39%  4 | 8.47%  10 | 10.17%  12 | 29.66%  35 | 118 | 4.31 |
| Reduction in aortic dissections | 11.67%  14 | 16.67%  20 | 11.67%  14 | 3.33%  4 | 4.17%  5 | 9.17%  11 | 26.67%  32 | 16.67%  20 | 120 | 4.11 |
| Earlier intervention on aortic aneurysms | 6.72%  8 | 13.45%  16 | 17.65%  21 | 5.88%  7 | 6.72%  8 | 24.37%  29 | 13.45%  16 | 11.76%  14 | 119 | 4.22 |
| More families accessing genetic testing | 8.55%  10 | 10.26%  12 | 15.38%  18 | 27.35%  32 | 23.93%  28 | 6.84%  8 | 4.27%  5 | 3.42%  4 | 117 | 4.97 |
| More families accessing imaging surveillance | 6.67%  8 | 9.17%  11 | 14.17%  17 | 25.83%  31 | 24.17%  29 | 10.83%  13 | 5.83%  7 | 3.33%  4 | 120 | 4.76 |
| More effective control of blood pressure | 4.27%  5 | 8.55%  10 | 21.37%  25 | 11.11%  13 | 15.38%  18 | 24.79%  29 | 7.69%  9 | 6.84%  8 | 117 | 4.36 |
| Reduction in anxiety or depression | 13.93%  17 | 17.21%  21 | 6.56%  8 | 11.48%  14 | 11.48%  14 | 10.66%  13 | 22.95%  28 | 5.74%  7 | 122 | 4.58 |
| No reduction in quality of life as a result of screening | 21.67%  26 | 10.00%  12 | 6.67%  8 | 5.83%  7 | 9.17%  11 | 6.67%  8 | 9.17%  11 | 30.83%  37 | 120 | 4.18 |

Q42 If you participated in a trial of family screening, in addition to the information that would be collected as part of your standard care, would you be willing to send other details to clinicians and researchers, using a mobile phone app?

Answered: 131 Skipped: 42

| **ANSWER CHOICES** | **RESPONSES** |  |
| --- | --- | --- |
| Yes | 86.26% | 113 |
| No | 13.74% | 18 |
| TOTAL |  | 131 |

Q43 If you participated in a clinical trial of screening, how much time would you be willing to spend per week submitting data on a mobile phone app, to help the research? (minutes per week)

Answered: 118 Skipped: 55

| **#** | **RESPONSES** |
| --- | --- |
| 1 | An hour or 2 per week |
| 2 | 120 |
| 3 | 60 |
| 4 | 30 |
| 5 | 60 |
| 6 | 30 |
| 7 | I don't have apps |
| 8 | 30 |
| 9 | 60 |
| 10 | 20 |
| 11 | 15 |
| 12 | 30 |
| 13 | 60 |
| 14 | 30 |
| 15 | 30 |
| 16 | 0 |
| 17 | 30 |
| 18 | 10 |
| 19 | 07894 265357 |
| 20 | 15 |
| 21 | 60 |
| 22 | 60 |
| 23 | 20 |
| 24 | 10 |
| 25 | 45 |
| 26 | 20 |
| 27 | 60 |
| 28 | 60 |
| 29 | 20 mins |
| 30 | 30 |
| 31 | 1 hour |
| 32 | 60 |
| 33 | 60 |

| 34 | 20/30 |
| --- | --- |
| 35 | 60 |
| 36 | 70 |
| 37 | Whatever required |
| 38 | 20 |
| 39 | 60 |
| 40 | 10 |
| 41 | 10 |
| 42 | Whatever it takes |
| 43 | Not sure |
| 44 | 10 |
| 45 | 15 |
| 46 | 30 |
| 47 | 60 |
| 48 | 120 |
| 49 | 30 |
| 50 | 60 |
| 51 | no |
| 52 | 10 |
| 53 | 60 |
| 54 | 105 |
| 55 | 30 |
| 56 | 0 |
| 57 | 30 mins |
| 58 | 60 |
| 59 | 60 |
| 60 | 30 |
| 61 | 10 |
| 62 | 60 |
| 63 | 20 |
| 64 | 10 |
| 65 | 60 |
| 66 | 15 |
| 67 | 60 |
| 68 | 180 |
| 69 | 10 |
| 70 | 10 |
| 71 | 10 |
| 72 | As many as necessary |
| 73 | 30 |
| 74 | Any amount of time. |

| 75 | 60 |
| --- | --- |
| 76 | 60 |
| 77 | 30 |
| 78 | 60 |
| 79 | 30 |
| 80 | 30 |
| 81 | 60 |
| 82 | 60 |
| 83 | 60 |
| 84 | 60 |
| 85 | As much as needed |
| 86 | 30 |
| 87 | 15 |
| 88 | 30-60 mins a week |
| 89 | 30-60 |
| 90 | 30 |
| 91 | 15 |
| 92 | 30 |
| 93 | 20 |
| 94 | 10 |
| 95 | 30 |
| 96 | 60 |
| 97 | As much as needed |
| 98 | 60 |
| 99 | 15 mins per week |
| 100 | 20 |
| 101 | 30mi |
| 102 | 30 |
| 103 | 120 |
| 104 | 30 |
| 105 | 5 |
| 106 | 120 |
| 107 | 15minutes |
| 108 | 30 |
| 109 | 120 min |
| 110 | 30 |
| 111 | 60 |
| 112 | 60 |
| 113 | 15 |
| 114 | 60 |
| 115 | 120 |

| 116 | 60 |
| --- | --- |
| 117 | 30 |
| 118 | 45 |

Q44 How far you would be willing to travel to undergo screening? (miles from home)

Answered: 124 Skipped: 49

| **#** | **RESPONSES** |
| --- | --- |
| 1 | 200 |
| 2 | 200 |
| 3 | 20 miles |
| 4 | 50 |
| 5 | 50 |
| 6 | 20 |
| 7 | 300 |
| 8 | ten |
| 9 | 20 |
| 10 | 50 |
| 11 | 30 |
| 12 | 15 |
| 13 | 100 |
| 14 | 30 |
| 15 | 40 |
| 16 | 15 |
| 17 | 20 |
| 18 | 60 |
| 19 | 20 |
| 20 | 100 |
| 21 | 25 miles |
| 22 | 10 |
| 23 | 25 |
| 24 | 40 |
| 25 | 50 |
| 26 | 5 |
| 27 | 20 |
| 28 | 12 |
| 29 | 30 |
| 30 | 100 |
| 31 | 50 |
| 32 | 25 |
| 33 | 120 |
| 34 | 25 |

| 35 | 30 |
| --- | --- |
| 36 | 200 |
| 37 | 30 |
| 38 | 20 |
| 39 | 100 |
| 40 | 50 |
| 41 | 2 |
| 42 | Don’t mind how far |
| 43 | 10 |
| 44 | 60 |
| 45 | 8 |
| 46 | 20 |
| 47 | I will go anywhere if it helps |
| 48 | Not sure |
| 49 | 5 |
| 50 | 10 |
| 51 | 10 |
| 52 | 10 |
| 53 | 200 |
| 54 | 29 |
| 55 | 60 |
| 56 | 100 |
| 57 | 20 |
| 58 | 100 |
| 59 | 30 |
| 60 | 200 |
| 61 | 100 |
| 62 | Kent to London |
| 63 | 5 |
| 64 | 100 |
| 65 | I would travel nationally |
| 66 | 40 ish |
| 67 | 40 |
| 68 | 200 |
| 69 | 10 |
| 70 | 30 |
| 71 | 100 |
| 72 | 20 |
| 73 | 200 |
| 74 | 5 |
| 75 | 10 |

| 76 | 30 |
| --- | --- |
| 77 | 5 |
| 78 | Not sure - about 50/100 miles |
| 79 | 3 |
| 80 | 40 |
| 81 | 0 |
| 82 | I don’t mind |
| 83 | Within 30 miles radius |
| 84 | 30 |
| 85 | 30 |
| 86 | 5 |
| 87 | 200 |
| 88 | 50 |
| 89 | 50 |
| 90 | 200 |
| 91 | 100 |
| 92 | 50 |
| 93 | 10 |
| 94 | Ireland to UK |
| 95 | 10-25 |
| 96 | 50 |
| 97 | 5 |
| 98 | 100 |
| 99 | 15 |
| 100 | 2 |
| 101 | 20 |
| 102 | I’d travel anywhere! |
| 103 | 70 miles |
| 104 | 30 |
| 105 | 50 miles if mileage paid for, 15 if not |
| 106 | 200 |
| 107 | 40 miles |
| 108 | 30 |
| 109 | 150 |
| 110 | 30 |
| 111 | 50 |
| 112 | 20 |
| 113 | 5 miles |
| 114 | 100 |
| 115 | 30 |
| 116 | Any |

| 117 | Whatever it took |
| --- | --- |
| 118 | 15 |
| 119 | 30 |
| 120 | 30 |
| 121 | 100 |
| 122 | 10 |
| 123 | 70 |
| 124 | 100 |

Q45 Around half of the people who are screened for a genetic cause of aortic disease have an inconclusive result, one which cannot yet be interpreted with our current knowledge. This is known as a 'Variant of Uncertain Significance' or 'VUS'. Although no changes to the care of the patient and family follow from such a test, the result would need to be reassessed in future, as our knowledge grows. If the result of your family's genetic testing was inconclusive, how distressed do you think you would you feel, on a scale of 1-10? (Move the slider: 0=not at all distressed, 100=very distressed).

Answered: 126 Skipped: 47

|  | **AVERAGE NUMBER** |  |  |  | **RESPONSES** |  |
| --- | --- | --- | --- | --- | --- | --- |
|  |  | 33 |  |  |  | 126 |
|  |  |  |  |  |  |  |

Q46 Is your or your family’s care managed by a clinic that specialises in screening, diagnosing, counselling and treating patients with inherited cardiac conditions (sometimes known as an Inherited Cardiac Conditions or ICC centre)?

Answered: 126 Skipped: 47

| **ANSWER CHOICES** | **RESPONSES** |  |
| --- | --- | --- |
| Yes | 12.70% | 16 |
| No | 48.41% | 61 |
| I don't know | 38.89% | 49 |
| TOTAL |  | 126 |

Q47 Which of these people should have access to the Decision Support Tool, if it was to include your individual health data? (select all that apply)

Answered: 125 Skipped: 48

0

%

10

%

20

%

30

%

40

%

50

%

60

%

70

%

80

%

90

%

100

%

Me, as the

patient

My relatives

Surgeons

Cardiologists

Clinical

Geneticists

Aortic Nurses

GP

Other (please

specify)

Q48 If you & your family used a Decision Support Tool, would you be concerned about the possibility that other family members may learn of your test results indirectly through the Decision Support Tool?

Answered: 126 Skipped: 47

| **ANSWER CHOICES** | **RESPONSES** |  |
| --- | --- | --- |
| Yes | 28.57% | 36 |
| No | 71.43% | 90 |
| TOTAL |  | 126 |

Q49 Which of the following was your GP able to provide you with information about after your relative's aortic dissection (tick all that apply):

Answered: 124 Skipped: 49

| **ANSWER CHOICES** | **RESPONSES** |  |
| --- | --- | --- |
| Levels of physical activity that you can/should have | 6.45% | 8 |
| General lifestyle advice | 12.10% | 15 |
| Medications that you should take. | 6.45% | 8 |
| Tests that you should undergo | 16.13% | 20 |
| Implications for your family | 5.65% | 7 |
| None of the above | 79.03% | 98 |
| Total Respondents: 124 |  |  |

Q50 In general, do you feel that you were provided with adequate information and included in decision-making about your future care, as the relative of someone who had an aortic dissection?

Answered: 125 Skipped: 48w

| **ANSWER CHOICES** | **RESPONSES** |  |
| --- | --- | --- |
| Yes | 16.00% | 20 |
| No | 75.20% | 94 |
| I prefer not to say | 8.80% | 11 |
| TOTAL |  | 125 |

Q51 Which of the following medical specialists have been involved in your or your family's care for aortic disease? (tick all that apply)

Answered: 112 Skipped: 61

| **ANSWER CHOICES** | **RESPONSES** |  |  |
| --- | --- | --- | --- |
| General Practitioner | 68.75% |  | 77 |
| Cardiac Surgeons | 62.50% |  | 70 |
| Vascular Surgeons | 40.18% |  | 45 |
| Cardiologists | 66.96% |  | 75 |
| Clinical Geneticists | 34.82% |  | 39 |
| Radiologists | 28.57% |  | 32 |
| Psychologists | 6.25% |  | 7 |
| Aortic Nurses | 38.39% |  | 43 |
| Other (specify: ____) | 6.25% |  | 7 |
| Total Respondents: 112 |  |  |  |

Q52 Was there effective communication between the different specialist groups involved in you & your family's care?

Answered: 124 Skipped: 49

| **ANSWER CHOICES** | **RESPONSES** |  |
| --- | --- | --- |
| Yes | 18.55% | 23 |
| No | 33.06% | 41 |
| It varies | 39.52% | 49 |
| Prefer not to say | 8.87% | 11 |
| TOTAL |  | 124 |

Q53 Follow-up of aortic patients might require the involvement of different specialist clinical groups across different hospitals and NHS Trusts. Was this ever a source of problems, in your personal experience?

Answered: 123 Skipped: 50

| **ANSWER CHOICES** | **RESPONSES** |  |
| --- | --- | --- |
| Yes | 24.39% | 30 |
| No | 60.98% | 75 |
| Prefer not to say | 14.63% | 18 |
| TOTAL |  | 123 |

Q54 How familiar are you with using digital devices such as mobile phones, iPads or computers)?

Answered: 126 Skipped: 47

|  | **VERY FAMILIAR** | **SOMEWHAT FAMILIAR** | **NOT FAMILIAR** | **TOTAL** | **WEIGHTED AVERAGE** |
| --- | --- | --- | --- | --- | --- |
| Mobile Phone | 87.90%  109 | 10.48%  13 | 1.61%  2 | 124 | 1.14 |
| Tablet (e.g. iPad) | 83.87%  104 | 8.06%  10 | 8.06%  10 | 124 | 1.24 |
| Computer | 84.80%  106 | 14.40%  18 | 0.80%  1 | 125 | 1.16 |

Q55 Where do you most often go for medical information in your everyday life?

Answered: 124 Skipped: 49

| **ANSWER CHOICES** | **RESPONSES** |  |  |
| --- | --- | --- | --- |
| Consult with clinicians. | 27.42% |  | 34 |
| Ask other patients. | 0.81% |  | 1 |
| The Internet. | 64.52% |  | 80 |
| The Media. | 0.00% |  | 0 |
| Other (please specify) | 7.26% |  | 9 |
| TOTAL |  |  | 124 |

Q56 Which of the following information do you think patients and their families would benefit from knowing when getting involved in screening?

(tick all that apply)

Answered: 123 Skipped: 50

| **ANSWER CHOICES** | **RESPONSES** | |
| --- | --- | --- |
| Information about the disease for the participant, their family and their primary health care providers (e.g. GP) | 92.68% | 114 |
| Information about the screening tests to be performed | 91.87% | 113 |
| The implications of a positive genetic test (where something is found) | 91.87% | 113 |
| The implications of a negative genetic test (where nothing is found) | 69.92% | 86 |
| The implications of an uncertain genetic test | 77.24% | 95 |
| What happens when there is no clear diagnosis | 79.67% | 98 |
| When and how often imaging surveillance will occur | 80.49% | 99 |
| Information about lifestyle advice and medication that might be given for prevention | 80.49% | 99 |
| Reasons for any proposed treatment that might be offered | 75.61% | 93 |
| Information about potential future surgery | 75.61% | 93 |
| Total Respondents: 123 |  |  |

Q57 How would you prefer to receive the information required for shared decision making about screening?

Answered: 125 Skipped: 48

| **ANSWER CHOICES** | **RESPONSES** |  |  |
| --- | --- | --- | --- |
| A booklet | 29.60% |  | 37 |
| A mobile phone app | 31.20% |  | 39 |
| A website | 32.00% |  | 40 |
| A video | 4.80% |  | 6 |
| Other (please specify) | 2.40% |  | 3 |
| TOTAL |  |  | 125 |
| **#** | **OTHER (PLEASE SPECIFY)** | **DATE** | |
| 1 | No where | 2/3/2023 4:24 PM | |
| 2 | Email | 2/3/2023 7:38 AM | |
| 3 | Any of the above | 2/1/2023 6:57 AM | |

Q58 Which of the following information is most important for a family like yours affected by aortic dissection? [Rate each item]

Answered: 124 Skipped: 49

|  | **UNIMPORTANT** | **NICE**  **TO**  **HAVE** | **IMPORTANT** | **VERY**  **IMPORTANT** | **ESSENTIAL** | **TOTAL** | **WEIGHTED AVERAGE** |
| --- | --- | --- | --- | --- | --- | --- | --- |
| Understanding the possible diagnosis | 0.00%  0 | 2.42%  3 | 16.94%  21 | 27.42%  34 | 53.23%  66 | 124 | 4.31 |
| Understanding the possible results of imaging tests | 0.00%  0 | 1.64%  2 | 27.05%  33 | 31.15%  38 | 40.16%  49 | 122 | 4.10 |
| Understanding the possible results of a genetic test | 0.00%  0 | 0.81%  1 | 20.16%  25 | 33.87%  42 | 45.16%  56 | 124 | 4.23 |
| What does a positive imaging test mean for the patient? | 0.00%  0 | 0.81%  1 | 26.02%  32 | 28.46%  35 | 44.72%  55 | 123 | 4.17 |
| What does a negative imaging test mean for the patient? | 0.81%  1 | 4.84%  6 | 36.29%  45 | 27.42%  34 | 30.65%  38 | 124 | 3.82 |
| How will the results of the imaging test inform future care? | 0.00%  0 | 4.84%  6 | 25.81%  32 | 32.26%  40 | 37.10%  46 | 124 | 4.02 |
| What does a positive genetic test mean for the patient? | 0.00%  0 | 0.81%  1 | 25.20%  31 | 21.14%  26 | 52.85%  65 | 123 | 4.26 |
| What does a negative genetic test mean for the patient? | 1.64%  2 | 4.10%  5 | 36.07%  44 | 24.59%  30 | 33.61%  41 | 122 | 3.84 |
| How will the results of the genetic test inform future care? | 0.00%  0 | 2.42%  3 | 26.61%  33 | 30.65%  38 | 40.32%  50 | 124 | 4.09 |
| How frequent will tests be? | 0.81%  1 | 12.20%  15 | 26.02%  32 | 33.33%  41 | 27.64%  34 | 123 | 3.75 |
| What are the treatment options? | 0.00%  0 | 2.42%  3 | 16.94%  21 | 32.26%  40 | 48.39%  60 | 124 | 4.27 |
| What are the consequences of treatment? | 0.00%  0 | 3.23%  4 | 16.13%  20 | 29.84%  37 | 50.81%  63 | 124 | 4.28 |
| How do we know if treatment is working/will work? | 0.00%  0 | 4.03%  5 | 19.35%  24 | 29.84%  37 | 46.77%  58 | 124 | 4.19 |
| If the results of screening are inconclusive, what happens next? | 0.00%  0 | 6.56%  8 | 31.97%  39 | 28.69%  35 | 32.79%  40 | 122 | 3.88 |
| Other: … | 0.00%  0 | 0.00%  0 | 0.00%  0 | 0.00%  0 | 0.00%  0 | 0 | 0.00 |

Q59 If we require further input from patients and relatives, would you be willing to be contacted in future with requests to help our research activities to develop a Decision Support Tool?

Answered: 125 Skipped: 48

| **ANSWER CHOICES** | **RESPONSES** |  |
| --- | --- | --- |
| Yes | 92.00% | 115 |
| No | 8.00% | 10 |
| TOTAL |  | 125 |

Q60 If you were to become involved in helping us with this research, how would you prefer to do this? (select your 1st preference)

Answered: 121 Skipped: 52

| **ANSWER CHOICES** | **RESPONSES** |  |
| --- | --- | --- |
| Face-to-face meeting | 11.57% | 14 |
| Telephone call | 19.83% | 24 |
| Video call | 19.01% | 23 |
| E-mail | 49.59% | 60 |
| TOTAL |  | 121 |

## Standards for Reporting Qualitative Research (SRQR)*

<http://www.equator>-[network.org/reporting-guidelines/srqr/](http://www.equator-network.org/reporting-guidelines/srqr/)

**Page/line no(s).**

Title and abstract

| **Title** - Concise description of the nature and topic of the study Identifying the study as qualitative or indicating the approach (e.g., ethnography, grounded theory) or data collection methods (e.g., interview, focus group) is recommended | 1 |
| --- | --- |
| **Abstract** - Summary of key elements of the study using the abstract format of the intended publication; typically includes background, purpose, methods, results, and conclusions | 2 |

Introduction

| **Problem formulation** - Description and significance of the problem/phenomenon studied; review of relevant theory and empirical work; problem statement | 3 |
| --- | --- |
| **Purpose or research question** - Purpose of the study and specific objectives or questions | 3 |

Methods

| **Qualitative approach and research paradigm** - Qualitative approach (e.g., ethnography, grounded theory, case study, phenomenology, narrative research) and guiding theory if appropriate; identifying the research paradigm (e.g., postpositivist, constructivist/ interpretivist) is also recommended; rationale** | 3, 4, 5 |
| --- | --- |
| **Researcher characteristics and reflexivity** - Researchers’ characteristics that may influence the research, including personal attributes, qualifications/experience, relationship with participants, assumptions, and/or presuppositions; potential or actual interaction between researchers’ characteristics and the research questions, approach, methods, results, and/or transferability | 3, 4, 5 |
| **Context** - Setting/site and salient contextual factors; rationale** | 3, 4, 5 |
| **Sampling strategy** - How and why research participants, documents, or events were selected; criteria for deciding when no further sampling was necessary (e.g., sampling saturation); rationale** | 3, 4, 5 |
| **Ethical issues pertaining to human subjects** - Documentation of approval by an appropriate ethics review board and participant consent, or explanation for lack thereof; other confidentiality and data security issues | 3, 4, 5 |
| **Data collection methods** - Types of data collected; details of data collection procedures including (as appropriate) start and stop dates of data collection and analysis, iterative process, triangulation of sources/methods, and modification of procedures in response to evolving study findings; rationale** | 3, 4, 5 |
| **Data collection instruments and technologies** - Description of instruments (e.g., interview guides, questionnaires) and devices (e.g., audio recorders) used for data collection; if/how the instrument(s) changed over the course of the study | 3, 4, 5 |
| **Units of study** - Number and relevant characteristics of participants, documents, or events included in the study; level of participation (could be reported in results) | 3, 4, 5 |
| **Data processing** - Methods for processing data prior to and during analysis, including transcription, data entry, data management and security, verification of data integrity, data coding, and anonymization/de-identification of excerpts | 3, 4, 5 |
| **Data analysis** - Process by which inferences, themes, etc., were identified and developed, including the researchers involved in data analysis; usually references a specific paradigm or approach; rationale** | 3, 4, 5 |
| **Techniques to enhance trustworthiness** - Techniques to enhance trustworthiness and credibility of data analysis (e.g., member checking, audit trail, triangulation); rationale** | 3, 4, 5 |

Results/findings

| **Synthesis and interpretation** - Main findings (e.g., interpretations, inferences, and themes); might include development of a theory or model, or integration with prior research or theory | 5, 6, 7, 8, 9, 10 |
| --- | --- |
| **Links to empirical data** - Evidence (e.g., quotes, field notes, text excerpts, photographs) to substantiate analytic findings | 5, 6, 7, 8, 9, 10, Table 2, Table 3 |

Discussion

| **Integration with prior work, implications, transferability, and contribution(s) to the field -** Short summary of main findings; explanation of how findings and conclusions connect to, support, elaborate on, or challenge conclusions of earlier scholarship; discussion of scope of application/generalizability; identification of unique contribution(s) to scholarship in a discipline or field | 10, 11, 12 |
| --- | --- |
| **Limitations** - Trustworthiness and limitations of findings | 10, 11, 12 |

Other

| **Conflicts of interest** - Potential sources of influence or perceived influence on study conduct and conclusions; how these were managed | 1 |
| --- | --- |
| **Funding** - Sources of funding and other support; role of funders in data collection, interpretation, and reporting | 1 |

**Reference:**

O'Brien BC, Harris IB, Beckman TJ, Reed DA, Cook DA. Standards for reporting qualitative research: a synthesis of recommendations. Academic Medicine, Vol. 89, No. 9 / Sept 2014 DOI: 10.1097/ACM.0000000000000388
